# EMG-Assisted Muscle Force Driven Finite Element Model of the Knee Joint with Fibril-Reinforced Poroelastic Cartilages and Menisci

**DOI:** 10.1038/s41598-020-59602-2

**Published:** 2020-02-20

**Authors:** A. Esrafilian, L. Stenroth, M. E. Mononen, P. Tanska, J. Avela, R. K. Korhonen

**Affiliations:** 1 Department of Applied Physics, University of Eastern Finland, Kuopio, Finland; 2 NeuroMuscular Research Center, Unit of Biology of Physical Activity, Faculty of Sport and Health Sciences, University of Jyväskylä, Jyväskylä, Finland

**Keywords:** Biomedical engineering, Mechanical engineering

## Abstract

Abnormal mechanical loading is essential in the onset and progression of knee osteoarthritis. Combined musculoskeletal (MS) and finite element (FE) modeling is a typical method to estimate load distribution and tissue responses in the knee joint. However, earlier combined models mostly utilize static-optimization based MS models and muscle force driven FE models typically use elastic materials for soft tissues or analyze specific time points of gait. Therefore, here we develop an electromyography-assisted muscle force driven FE model with fibril-reinforced poro(visco)elastic cartilages and menisci to analyze knee joint loading during the stance phase of gait. Moreover, since ligament pre-strains are one of the important uncertainties in joint modeling, we conducted a sensitivity analysis on the pre-strains of anterior and posterior cruciate ligaments (ACL and PCL) as well as medial and lateral collateral ligaments (MCL and LCL). The model produced kinematics and kinetics consistent with previous experimental data. Joint contact forces and contact areas were highly sensitive to ACL and PCL pre-strains, while those changed less cartilage stresses, fibril strains, and fluid pressures. The presented workflow could be used in a wide range of applications related to the aetiology of cartilage degeneration, optimization of rehabilitation exercises, and simulation of knee surgeries.

## Introduction

Knee osteoarthritis (KOA) is a multifactorial, chronic joint disease with high health-related costs^1^. It limits physical activities and reduces the quality of life by reducing the joint range of motion, destabilizing the joint, weakening muscles, and is associated with unbearable pain^2^. Several studies have emphasized that altered mechanical loading of the knee joint contributes to the onset and progression of KOA^3,4^ and affects directly the knee pain caused by KOA^5^. Muscle forces have been reported to be the largest contributors to the tibiofemoral joint contact force (JCF)^6^. Direct measurement of the JCF requires placing force measurement sensors into the knee joint. This highly invasive operation makes it unethical and practically difficult to implement in subjects^7–9^. Thus, alternative methods such as musculoskeletal (MS) and finite element (FE) modeling are highly desirable to simulate knee joint loading during different activities.

MS models estimate muscle forces and JCFs using kinematic and kinetic data obtained while performing an activity. Although MS models can estimate JCFs, they cannot assess tissue responses and parameters that may control articular cartilage degradation during the progression of KOA. FE modeling has been used for this purpose^10,11^. Thus far, numerous MS and FE models have been developed to evaluate knee joint loadings and stress distributions^6,9,12–26^. However, only a few studies have been conducted to combine subject-specific MS and FE models into a multiscale modeling workflow (from joint to tissue level) of the knee joint loading^11,14,18,23,27–29^.

Regarding the MS models, static-optimization^6,16,30^ and electromyography (EMG) assisted approaches^12,26,31^ are the main methods to estimate muscle activation levels and further muscle forces. In a static-optimization method, muscle activation levels are calculated according to the kinematics and kinetics, without considering subject-specific muscle activations^14,18,29,30,32^. Muscle activation and co-contraction levels could significantly vary in different activities and disorders such as KOA patients in comparison with healthy subjects despite small variations in kinematics and kinetics^33–35^. In these scenarios, previous studies^26,36–40^ have suggested that assisting the optimizer with EMGs improves the accuracy of the estimated muscle activations and the JCF. Different EMG-assisted MS models have been developed previously^12,26,38,40^.

In several previous FE models linked with MS models^11,14,19,27,41,42^, FE models were not driven directly by muscle forces. Thus, the total JCF (as a single force vector in 3 directions), as well as the joint moments, were directly applied on a single reference point of the femur in the FE model. Consequently, the measured knee moments were scaled in those studies by assuming that muscles generate most of these moments. This assumption alters the forces and moments which should be counterbalanced by ligaments and, as a result, could alter the joint secondary kinematics, kinetics, contact regions, and tissue mechanical responses^24,30,32,43^. In a muscle force driven FE model, on the contrary, the total joint moment is counterbalanced by the interaction of muscles and ligaments, which improves the subject specificity of the joint loading.

Several muscle force driven FE models have been developed earlier^18,23,24,28,29,32,44^. However, no studies have been conducted to combine an EMG-assisted MS model with a muscle force driven FE model. Furthermore, in some studies cartilages are limited to elastic material models and menisci are excluded, despite the crucial role of menisci in load distribution and stress concentration within cartilages^45,46^. Even though the incompressible elastic assumption for cartilage may provide an equivalent response with the poroelastic model in short-term loadings^47^, investigation of fluid flow and fluid pressure require poroelasticity. Inclusion of poroelasticity is essential since fluid can carry ~75% of the instantaneous load of cartilage^48^ and the fluid pressure is altered in early OA^49,50^. A fibril-reinforced poroviscoelastic (FRPVE) or poroelastic (FRPE) material considers a porous and hyperelastic media reinforced by collagen fibers^51^, which can estimate the contribution of different constituents (collagen, proteoglycans, fluid) on the mechanical response of the tissues. Those FE models that include these complex materials for soft tissues are not muscle force driven^11,14,20,27,41^.

Some muscle force driven FE models, again without EMG-assistance, have included fibril-reinforced hyperelastic composite material models for cartilages^23,24,28,32,44^. However, these studies^23,24,28,32,44^ did not analyze a continues gait cycle but statically analyzed specific time points during the stance phase of the gait. They also did not include poroelasticity or viscoelasticity of cartilage or menisci. Moreover, muscles were assumed to counterbalance the external abduction-adduction and internal-external moments in addition to the flexion-extension moment of the knee joint^23,24,28,32,44^. It has been suggested that this assumption results in overestimated muscle forces and JCFs^30,32^.

The estimated knee secondary kinematics, JCF, and soft tissue mechanical responses of a FE knee joint model can be affected by uncertainties in knee joint geometries, kinetics and primary kinematics, and material properties of tissues^52,53^. However, in a typical modeling approach, subject-specific geometries and primary/secondary kinematics can be obtained from MRI and motion analysis^54,55^. There have also been attempts to incorporate subject-specific material properties of cartilage from MRI into FE models^21,56,57^. On the contrary, ligament properties and specifically their pre-strains are challenging to be approximated, which cause uncertainties in modeling^58^. Several studies have aimed to represent the effect of ligament stiffness and pre-strain on the knee joint kinematics and contact parameters^59,60^ or tried to optimize the material properties based on experiments^61^. Nonetheless, it is still unclear how much ligament pre-strains can change contact parameters, especially stress, strain, and fluid pressure within the knee joint cartilage.

Therefore, this study aimed to develop an EMG-assisted MS model combined with a muscle force driven FE model of the knee joint with complex poroelastic material models for cartilage and menisci, and then evaluate the effect of ligament pre-strains on the knee kinematics, kinetics, contact area, and cartilage mechanical responses during the stance phase of gait. The main novelty of the study is the multiscale modeling workflow, including subject-specific EMG-based muscle activities, direct implementation of muscle forces in the FE model, continuous analysis of the whole stance phase of gait, and the FRPVE and FRPE material models for cartilages and menisci. Limitations of the previous studies and the novelties of the current study can also be seen at a glance in the supplementary material (Table S1).

## Methods

### Gait data and MS model

**Figure 1:**
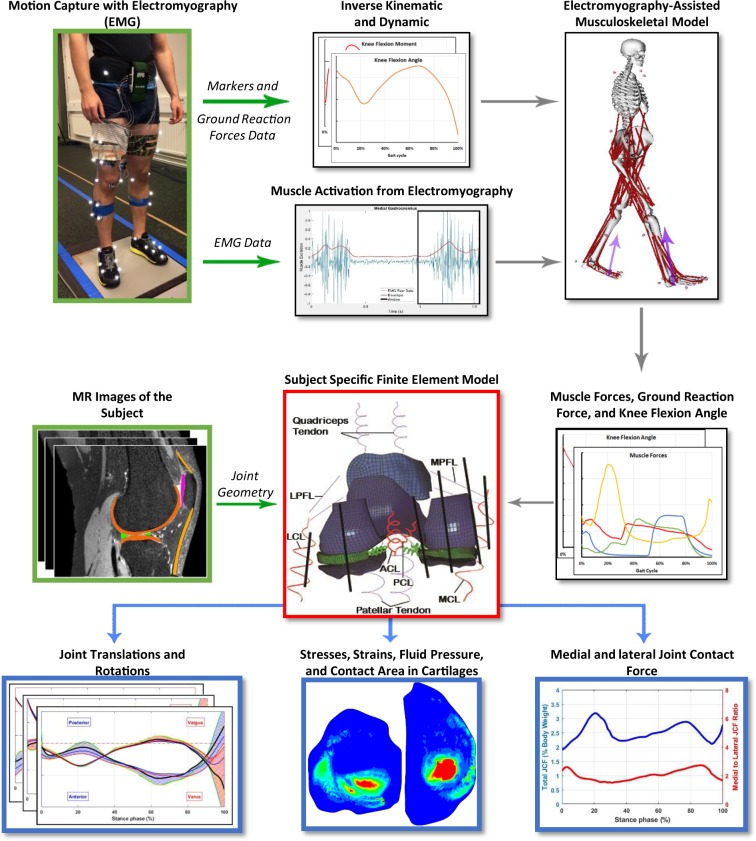
Workflow of the study. Green arrows indicate the input data and blue arrows illustrate results.

Figure 1 illustrates the workflow of this study. One healthy subject (male, 33 years old, 78 kg, 1.77 m) participated in experimental data collection. Five walking trials with the preferred speed of the subject were conducted at the gait analysis laboratory of the Faculty of Sport and Health Sciences, University of Jyväskylä, Finland. Marker trajectories (120 Hz, MX system, Vicon, UK), ground reaction forces (GRF, 1200 Hz, two force plates, OR6–6, AMTI, USA), and EMG signals (1200 Hz, Telemyo 2400T-G2, Noraxon, USA) were recorded from the trials. EMG signals were measured from vastus lateralis, rectus femoris, long head of biceps femoris, semitendinosus, medial gastrocnemius, soleus, and gluteus maximus during walking (more information in the supplementary material). The best trial was selected out of the five measured trials (in terms of the marker recognition, quality of EMG signals, and signal noise). In addition, magnetic resonance imaging (MRI) was performed using a clinical 3.0 T MRI system (Philips Healthcare, Best, The Netherlands). The data collection was done with the permission (94/2011) from the local ethical committee of the Kuopio University Hospital, Kuopio, Finland. The participant signed a written and informed consent and all the experiments were performed in accordance with relevant guidelines and regulations (principles set by the Declaration of Helsinki).

A standard 1 degree of freedom (DOF) Gait2392 MS model of the OpenSim (v.3.3, SimTK) software was utilized in this study^62^. One DOF knee model was deemed sufficient, since we used the MS model to estimate muscle forces, while secondary kinematics were estimated only by the FE model^6,29,30,63–68^. Muscle-tendon units were modeled as the Hill’s muscle model including elastic tendons. The geometry, mass and inertial properties, as well as muscle properties which depend on length (such as optimal fiber length and tendon slack length) of the MS model, were scaled based on the static trial of the subject.

The primary kinematics of the knee joint (the flexion angle) was calculated using an optimization method. The optimization was used to minimize the error between the measured trajectory of the markers and the corresponding virtual markers on the MS model^62^. Different weights on each marker were tested (which must be done for every analysis in the OpenSim) to ensure that rational primary kinematics are estimated according to the gait of healthy subjects from literature. Then, the residual reduction algorithm (RRA) was used to make the estimated primary kinematics dynamically consistent with the measured kinetics (i.e. GRF)^62^.

The Computed Muscle Control (CMC) toolbox^69,70^ of the OpenSim software (with its default activation dynamics and force-velocity relationships of the muscles), assisted with EMGs, was used to estimate muscle forces. Previous studies have shown^39,71^ that assisting the static-optimization/CMC toolbox with EMGs estimates the JCF/muscle forces more consistent with experiments, compared to the static-optimization/CMC method without EMG assistance. Muscle lines of action, as well as effective moment arms, were extracted utilizing an OpenSim plugin^72^.

The CMC toolbox utilizes an optimization technique (static-optimization) as well as a closed-loop proportional-integral-derivative (PID) controller to estimate muscle forces while tracking the measured gait kinematics^69^. As a result, each muscle excitation can vary from 0.02 (considered as zero excitation) to 1 (fully excited) without any penalization factor^70^. Nonetheless, in an EMG-assisted MS model, a penalty (or a weight) factor forces the optimization algorithm to find each muscle excitation within a range of the measured EMG of the corresponding muscle. For those muscles without measured EMGs, any excitation level within the default range (0.02–1) is considered as an acceptable solution^73^. In summary, the muscle activations were found by: 1) minimizing the error between the external flexion-extension moment on the knee joint and the moment generated by muscles, 2) minimizing the estimated muscle activations, and 3) estimating the activation of the measured muscles within a specific range of the measured EMGs.

**Figure 2:**
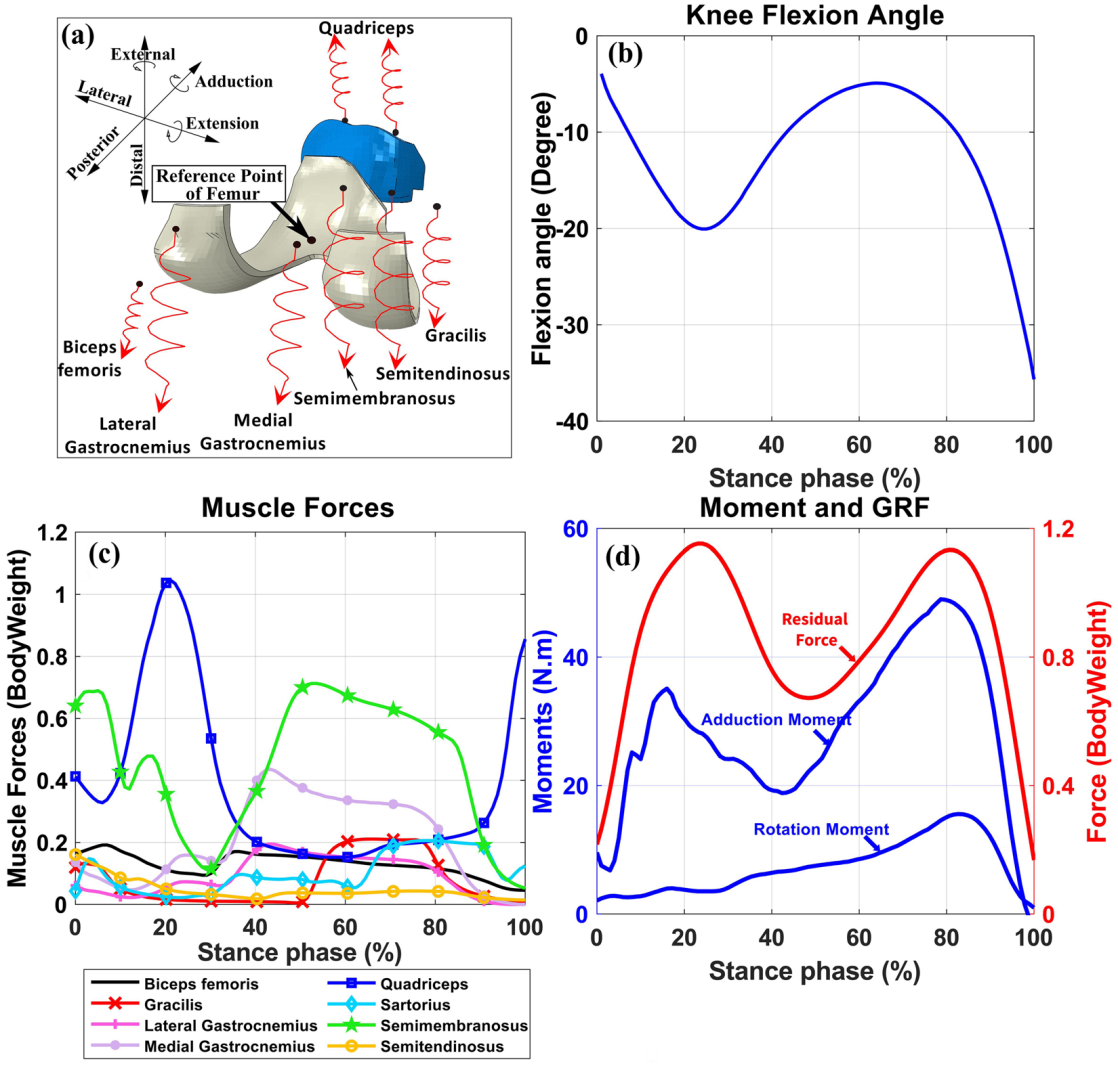
Inputs to the FE model. (**a**) Illustration of muscles in the FE model. Black spots on muscles were coupled to the femur’s reference point and red spots were free in space to apply force vectors. Springs were used for a better illustration of muscle line of actions and do not represent muscle fibers. (**b**) Knee flexion angle, (**c**) Muscle forces (magnitudes), and (**d**) Residual force passing through the knee joint, knee adduction and rotation moments (amplitudes).

We calculated normalized muscle activation levels from the EMG signal of the measured muscles and imported them into the CMC toolbox (more information on EMG measurements and analysis is presented in the supplementary material). Different ranges from zero (which uses the exact EMG signals as muscle activations) up to ±0.3 of normalized EMGs (which allows muscle activations to vary within ±0.3 unit of normalized measured EMGs) were tested to find the best range which could estimate muscle activations comparable to literature^18,74^ while minimizing the reserve actuators in the acceptable range. Eventually, a range of ±0.1 unit of normalized EMGs was selected to constrain the activation of muscles in the MS model. Consequently, enveloped EMG signals and corresponding muscle forces presented in Fig. 2C have the same patterns. The acceptable excitation range for the rest of the muscles was set to the default values of the CMC toolbox. In summary, the MS model was used to calculate the loading conditions as inputs to the FE model (Fig. 2, explained in detail in the next chapter).

### FE model

#### Geometry and material properties

The FE model geometry including femoral, tibial and patellar cartilage, and menisci exploited our formerly developed FE model^41^. Cartilages were modeled as a FRPVE material^75,76^ and menisci as a FRPE material^75,77,78^. Cartilages had the depth-dependent Benninghoff-type (arcade-like) architecture of collagen fibrils while the primary fibrils in meniscus were oriented circumferentially^79–83^. More information on segmentation, meshing, and material model is presented in the supplementary material.

Anterior cruciate ligament (ACL), posterior cruciate ligament (PCL), lateral collateral ligament (LCL), and medial collateral ligament (MCL) were modeled as nonlinear spring bundles^84^ including slack, toe and linear regions. The material parameters were adopted from a previous experimental study in which loading at high strain-rate (100% s^−1^)^85^ was applied on cadaver knees. Viscoelasticity was not explicitly employed since the effect of the viscous component of ligaments (stiffening with higher loading rate) at walking speed is implicitly considered in the nonlinear spring model. Furthermore, it has been shown that knee models with ligaments represented as elastic spring elements provide acceptable results^42^. More information on the selection of the constitutive model of knee ligaments is provided in the supplementary material (section 2.2.2).

Different fiber bundles of each ligament (for instance, anteriomedial and posteriolateral fiber bundles of the anterior cruciate ligament) were not modeled separately in this study since we could not distinguish those in MRIs. Therefore, average pre-strains of the fiber bundles of each ligament were assigned^84^. It should be mentioned that each ligament was modeled as a bundle of nonlinear spring elements. Due to different cross-sectional areas at the insertion points of each ligament, as estimated from MRI, ACL consisted of 60, PCL consisted of 100, LCL consisted of 15, and MCL consisted of 20 nonlinear spring elements (each element representing about 1 mm^2^ area). The force-strain relation at each ligament element was then formulated as follows:

1 $${\rm{f}}=\{\begin{array}{ll}0 & {\rm{\varepsilon }} < 0\\ \frac{1}{4}{{\rm{K}}}_{{\rm{l}}}{{\rm{\varepsilon }}}^{2}/{{\rm{\varepsilon }}}_{{\rm{l}}} & 0\le {\rm{\varepsilon }}\le 2{{\rm{\varepsilon }}}_{{\rm{l}}}\\ {{\rm{K}}}_{{\rm{l}}}({\rm{\varepsilon }}-{{\rm{\varepsilon }}}_{{\rm{l}}}) & {\rm{\varepsilon }} > 2{{\rm{\varepsilon }}}_{{\rm{l}}}\end{array}$$

where ${\rm{f}}$ is the tensile force in each ligament element, ${{\rm{K}}}_{{\rm{l}}}$ is the ligament stiffness (Table 1), ${{\rm{\varepsilon }}}_{{\rm{l}}}$ represents the end of the toe region and was set to 0.03^86^, and ${\rm{\varepsilon }}$ is the current strain in the ligament. Meniscal horn attachments were modeled as linear spring bundles^87^.

**Table 1:** Material parameters of ligaments used in the simulation (see Eq. 1).

Ligament Bundle	${{\rm{K}}}_{{\rm{l}}}$ (kN)	${{\boldsymbol{\varepsilon }}}_{{\bf{r}}}$ (%)				
		−10%	−5%	**Reference**	+5%	+10%
ACL	10	−2	3	**8**	13	18
PCL	18	−23.5	−18.5	**−13.5**	−8.5	−3.5
LCL	6	−17.3	−12.33	**−7.33**	−2.33	2.67
MCL	8.25	−6.34	−1.34	**3.66**	8.66	13.66

${{\rm{K}}}_{{\rm{l}}}$ is the linear stiffness and ${{\rm{\varepsilon }}}_{{\rm{r}}}$ is the ligament pre-strain for the fully extended knee joint^84^.

#### Loading, boundary conditions, and simulations

Each FE simulation, excluding the MS model analysis and FE model generation, took up to 30 hours on a typical CPU. Embedding boundary/loading conditions of the MS model into the FE model to include simultaneous muscle force estimations brings an iterative process due to the existence of the secondary kinematics, soft tissue deformations, etc^18,29^. As a result, the embedded model increases the simulation time considerably and brings convergence difficulties to the FE model (e.g. due to the highly nonlinear FRPVE material model). Thus, the MS model and the FE model were implemented in series.

Loading and boundary conditions of the FE model, obtained from the MS model (see above the MS model section), consisted of 1) the knee flexion angle, 2) the muscle force vectors for each considered muscle, 3) the residual forces passing through the knee joint, and 4) the knee abduction-adduction and internal-external moments (Fig. 2). All quantities, as well as the results of the study, are reported in the local coordinate system fixed to the proximal tibia (Fig. 2a shows the local coordinate system).

The resultant force at each muscle, which is the sum of the active and passive forces, was imported to the FE model (Fig. 2c). To keep the MS and FE models identical, muscle insertion points, as well as muscle moment arms, were imported from the MS model to the FE model. One end of each muscle (which was represented by spring element) was coupled to the reference point of the femur and the other end was free in space to apply the muscle force vector, including both the magnitude of the muscle force and its direction (Fig. 2a). The reference point of the femur was defined as the middle of the lateral and medial femoral condyles, and all the femoral nodes on the cartilage-bone interface were coupled to this reference point^20^. Consequently, muscles generated moments in the knee joint. Except for the flexion-extension angle, which was set to follow the primary kinematics, all other rotations and translations were then resisted by muscles and passively by ligaments. See the supplementary material for a detailed explanation of the loading conditions in the knee joint.

The residual forces passing through the knee joint (Fig. 2d) consisted of 1) the inertial forces due to the accelerations, and 2) the internal forces (excluding muscle forces) generated by the external forces (i.e. GRF). See supplementary material for more details on the residual forces. The residual force vector, external joint moments, and the knee flexion angle were first transformed to the local coordinate system of the FE model (Fig. 2a) and then were applied to the reference point of the femur.

We have developed a MATLAB script to read outputs from the MS model and update the loading/boundary conditions of the FE model, accordingly. Then, the script runs the FE model in Abaqus and finally extracts the results from the FE model. Both the femur and the patella had 6 DOFs (3 translations and 3 rotations) in the FE model. All the inputs to the FE model were transformed and presented in the local coordinate system of the FE model (femur relative to the tibia). Thus, the bottom of the tibial cartilage was fixed in all directions and the relative forces and movements were applied on the femur (Fig. 2). The initial condition of the FE model was set to heel strike and the complete stance phase of gait was simulated using soils consolidation analysis of the Abaqus software. Running of the whole workflow, excluding the model generation, took about 40 hours of an Intel E5-2690 CPU time, single-core analysis.

#### Ligament sensitivity analysis

Since the magnitude of the ligament pre-strain, in addition to muscle contributions, is one of the major uncertainties in the knee modeling and may significantly affect kinematics and the JCF on the joint surfaces^88–90^, we performed a sensitivity study of the effect of pre-strain in ACL, PCL, LCL, and MCL on kinematics, kinetics and tissue responses. Table 1 shows the range of the used pre-strain values. We selected wide pre-strain ranges to emphasize the effects of pre-strain uncertainties on the results, also including zero pre-strain in each ligament. The pre-strain values of the other three ligaments were set to the reference values, while only the pre-strain of the ligament of interest was changed in each simulation.

## Results

### Joint contact force

**Figure 3:**
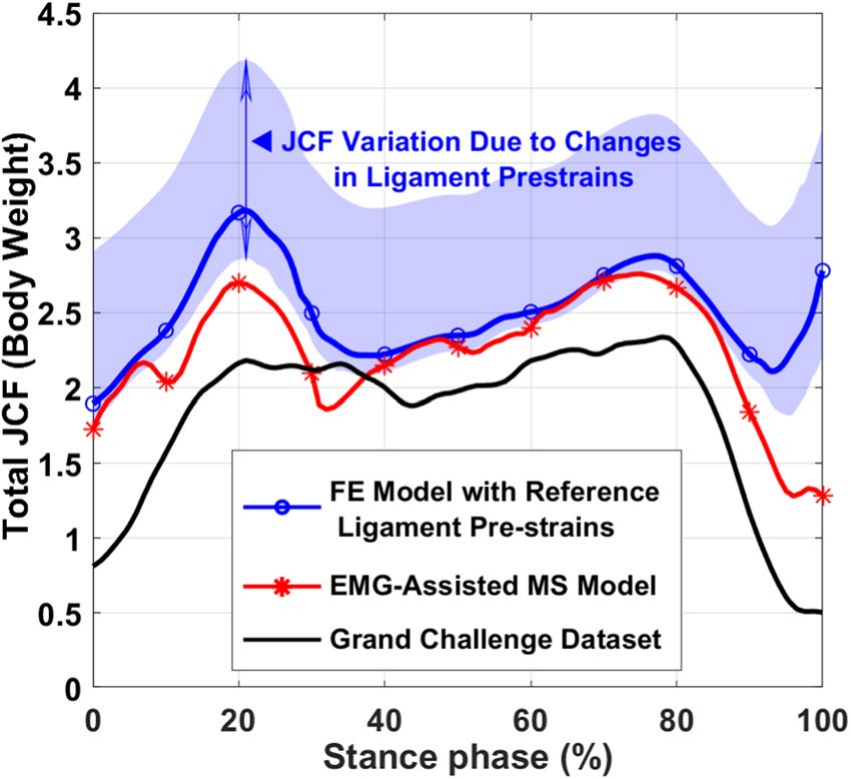
JCF calculated by the EMG-assisted MS model and the FE model compared with JCFs from the sixth grand challenge dataset^12^. The highlighted area (JCF variation) shows the maximum and minimum JCFs from all ligament sensitivity analysis simulations (ligament stiffnesses and pre-strains in Table 1). JCFs are given in the bodyweight of the subject over the stance phase.

Figure 3 illustrates JCFs calculated by the EMG-assisted MS model and the muscle force driven FE model of the study as well as the *in vivo* knee JCFs from the sixth grand challenge dataset (average of normal walking gait trials)^9^.

**Figure 4:**
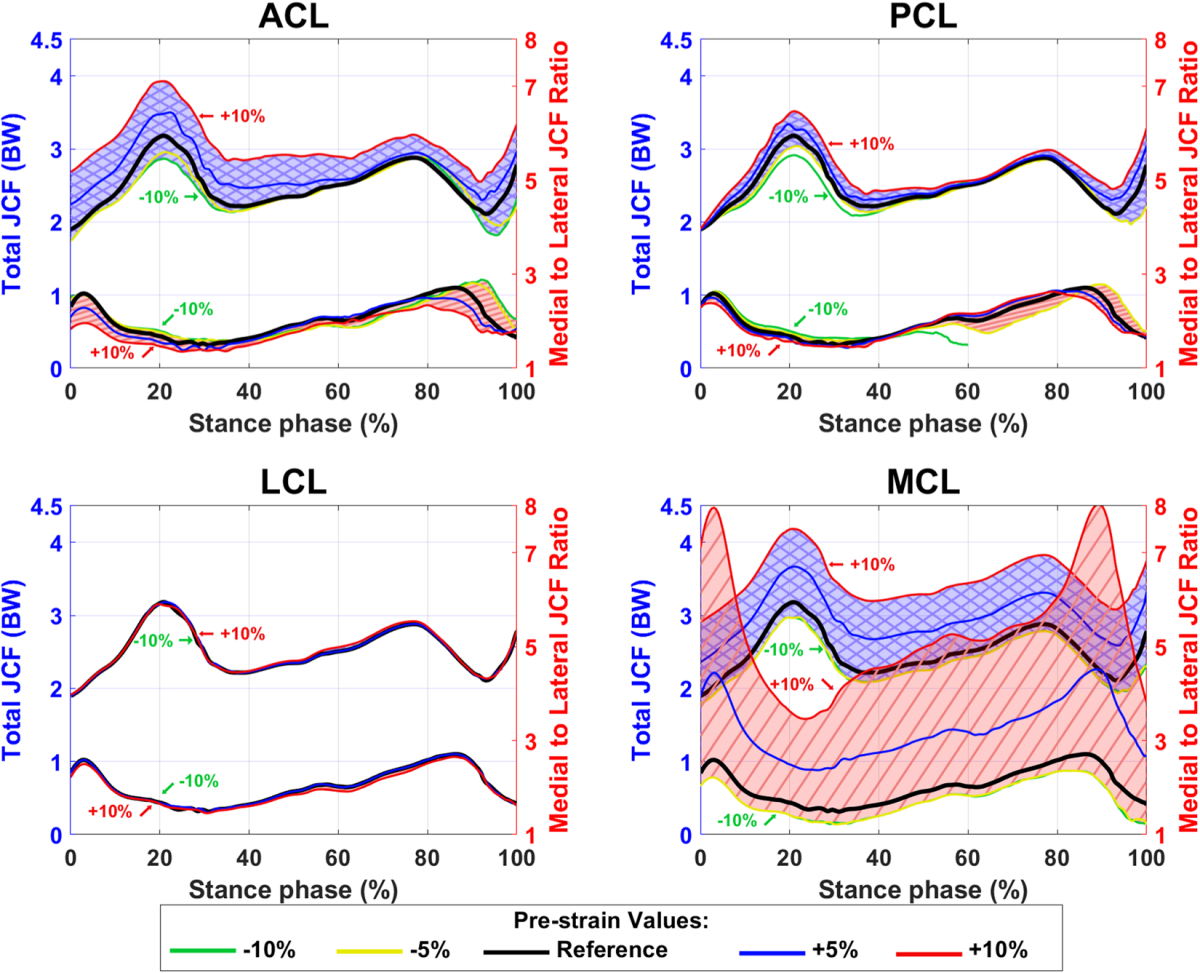
Total JCF with different pre-strains of ACL, PCL, LCL, and MCL bundles. The blue shaded areas (with crosslines) show the total JCF (left axis) and the red shaded areas (diagonal lines) show the medial to lateral ratio of JCF (right axes).

**Figure 5:**
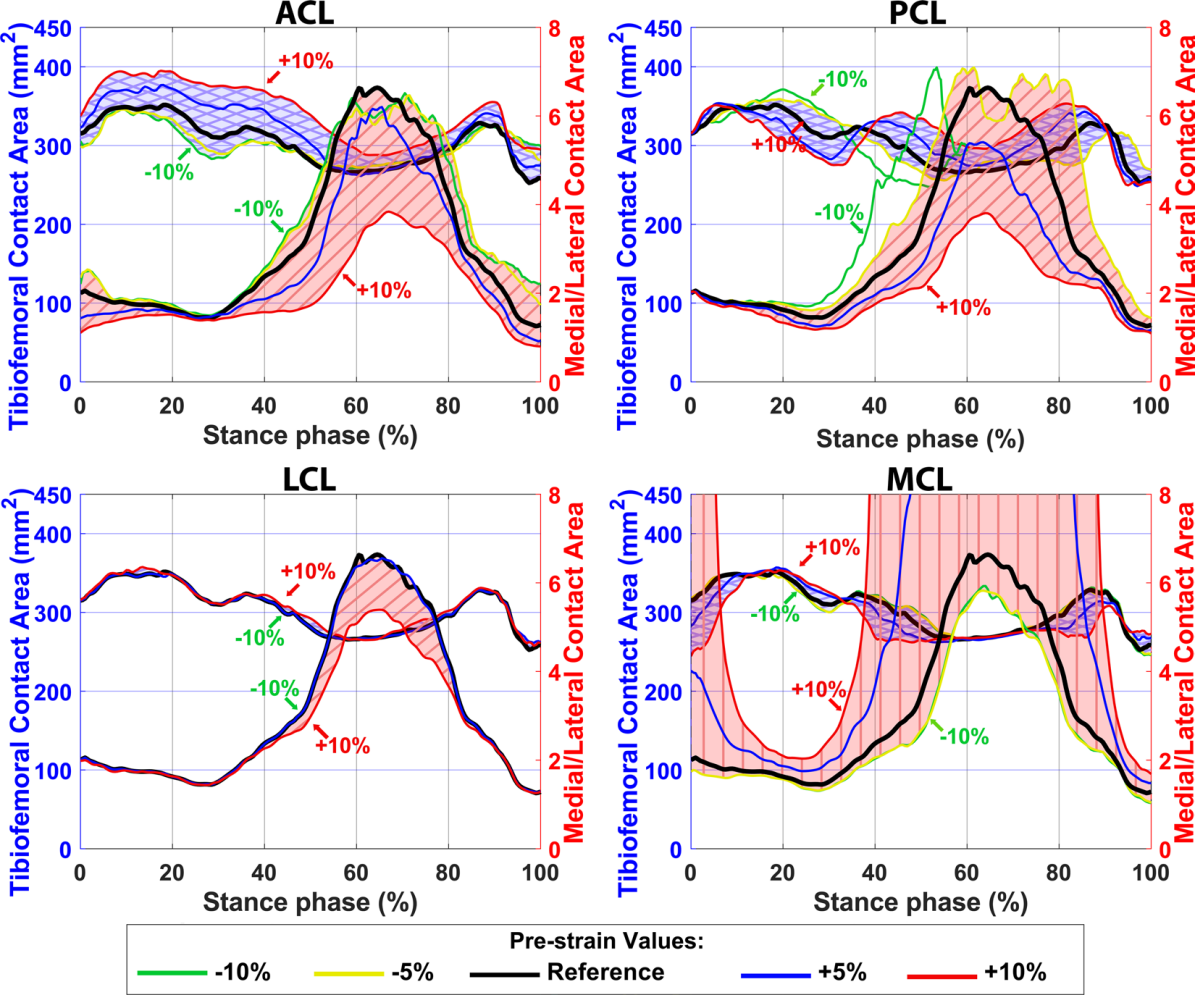
Contact areas between femoral and tibial cartilage with different pre-strains of ACL, PCL, LCL, and MCL bundles. The blue shaded areas (with crosslines) show the total contact area (left axis) and the red shaded areas (diagonal lines) show the medial cartilage to lateral cartilage ratio of contact area (right axes). The cartilage-to-cartilage contact area on the lateral side of the knee was close to zero in the FE models with 5–10% increased pre-strains in the MCL (at 40% to 90% of the stance phase). Thus, the medial to lateral contact area ratio goes out of range.

Sensitivity analysis indicates that higher pre-strain in ACL, PCL, and LCL increased the JCF and moved both the JCF distribution and the tibiofemoral contact area to the lateral side of the joint (Figs. 4 and 5). However, an increase in the MCL pre-strain led to a noticeable increase in the JCF with a distinctive relocation of the JCF distribution and the tibiofemoral contact area toward the medial side. Moreover, ligaments with pre-strains less than −5% of the reference values did not considerably affect the JCF.

### Joint kinematics

**Figure 6:**
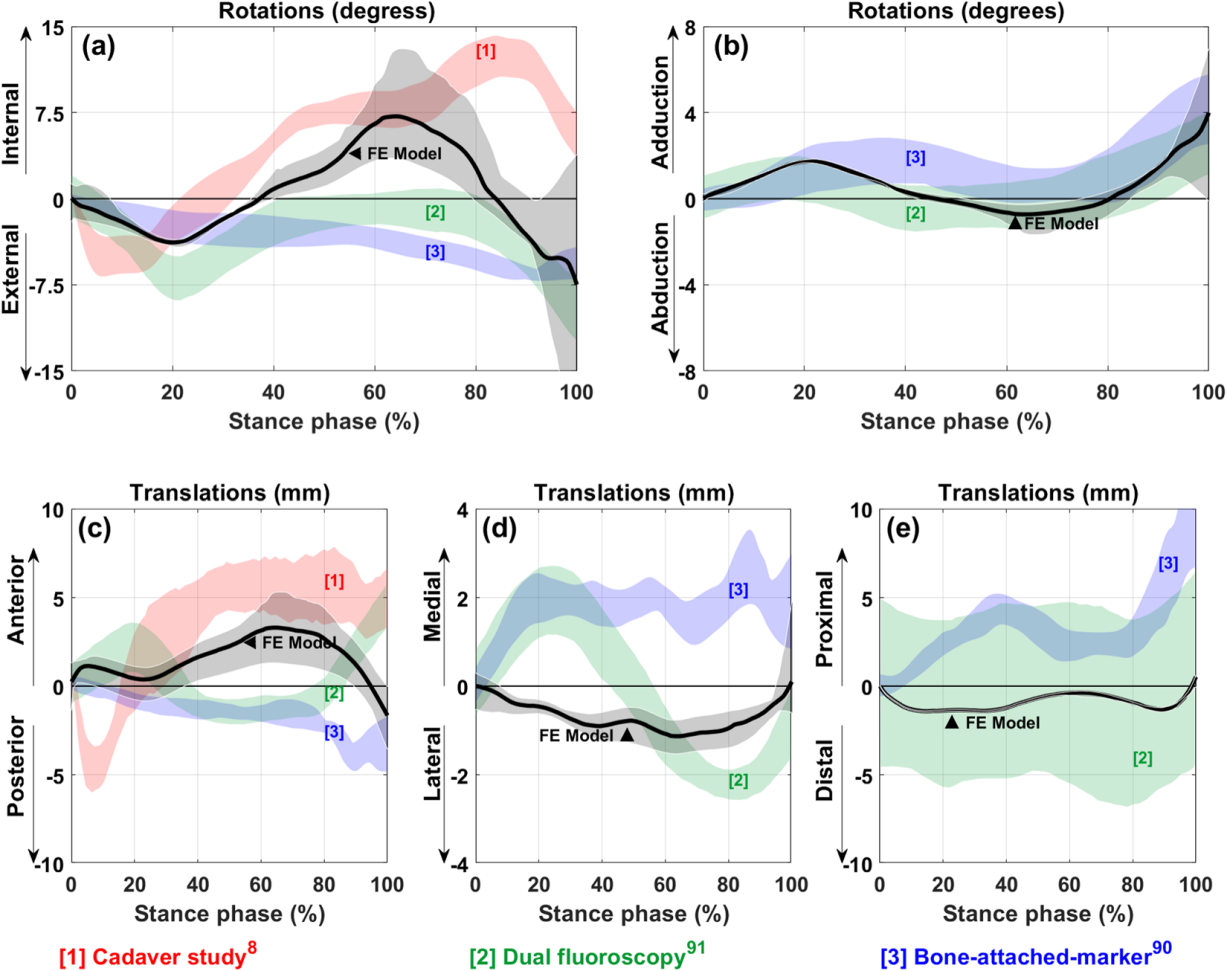
Secondary kinematics of the knee joint from FE model in comparison with experimental measurements. (**a**) Internal/External rotation, (**b**) Abduction/Adduction rotation, (**c**) Anterior/Posterior translation, (**d**) Medial/Lateral translation, and (**e**) Distal/Proximal translation of the femur relative to the tibia. The solid black line shows the results from FE model based on the reference pre-strains and the black shaded area represents changes in value due to the pre-strain changes. To make the comparison easier, all the graphs have been shifted to start from zero.

Secondary kinematics of the knee joint obtained from the FE model was compared with experimental results from a cadaver study^8^, bone-attached-marker gait analysis^91^, and gait analysis measured by fluoroscopic imaging system^92^ in Fig. 6.

**Figure 7:**
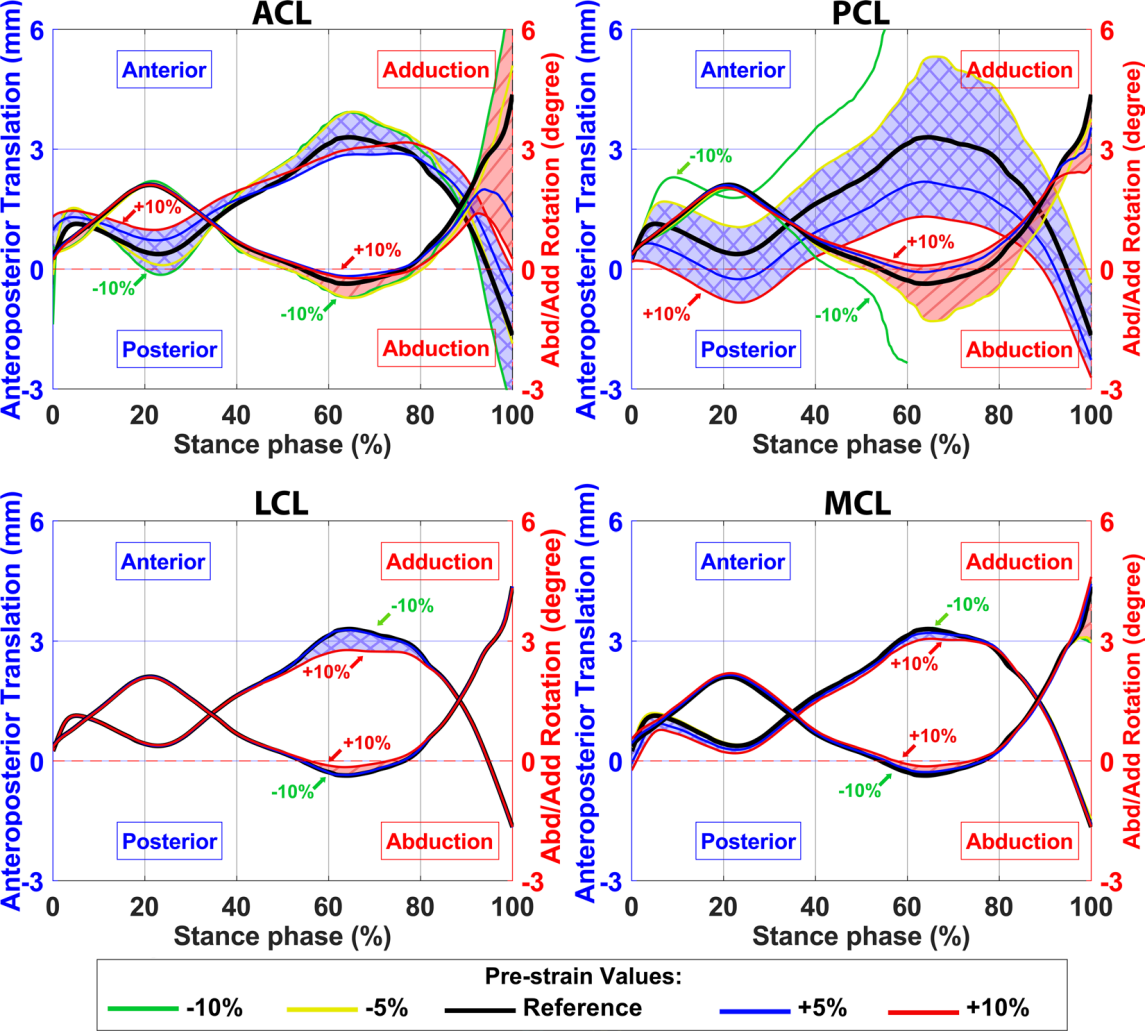
Secondary knee joint kinematic in anteroposterior direction calculated by the FE model with different pre-strains of ACL, PCL, LCL, and MCL bundles. The blue shaded areas (with crosslines) show the anterior/posterior translation of the femur with respect to the tibia (left axis) and the red shaded areas (diagonal lines) show the abduction/adduction rotation of the femur (right axes).

**Figure 8:**
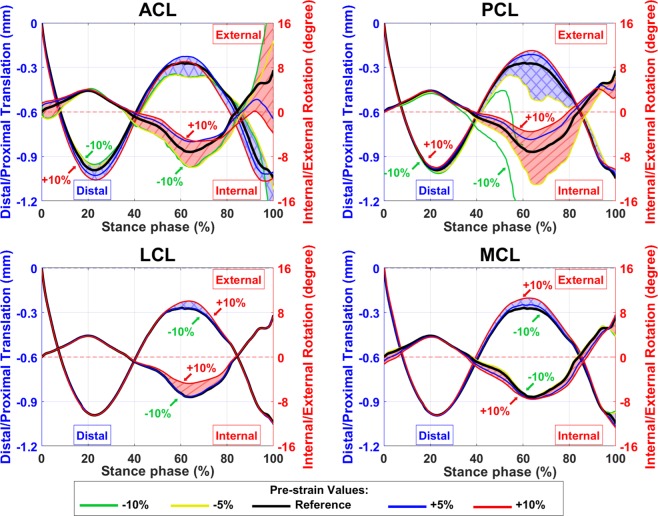
Secondary knee joint kinematic regarding the distal-proximal direction calculated by FE model with different pre-strains of ACL, PCL, LCL, and MCL bundles. The blue shaded areas (with crosslines) show the superior/inferior translation of the femur with respect to the tibia (left axis) and the red shaded areas (diagonal lines) show the internal/external rotation of the femur (right axes).

Secondary kinematics of the knee joint with different ligament pre-strains calculated by the FE model are shown in Figs. 7 and 8. Ligament pre-strains did not change the joint motion pattern but had a substantial influence on the range of motion of the secondary kinematics. Tightening the ACL shifted the femur forward (anterior direction) at the heel strike and toe-off, while it decreased the range of anteroposterior translation and internal rotation of the femur (Figs. 7 and 8). Moreover, higher pre-strain in ACL moved the femur toward the medial side of the joint during the stance phase and kept it at the joint center at the toe-off (supplementary material, Fig. S2).

The PCL pre-strain did not affect the position of the femur at the heel strike or toe-off in any direction. However, it influenced the range of motion of the secondary kinematics (Figs. 7, 8, and S2). Lowering the pre-strain of PCL below −5% of the reference value increased the joint range of motion up to excessive anterior translation as well as excessive abduction and internal rotations of the femur (Figs. 7 and 8, green lines). On the other hand, joint kinematics were not significantly altered by changing the pre-strain in LCL or MCL (Figs. 7 and 8).

### Cartilage tissue response

**Figure 9:**
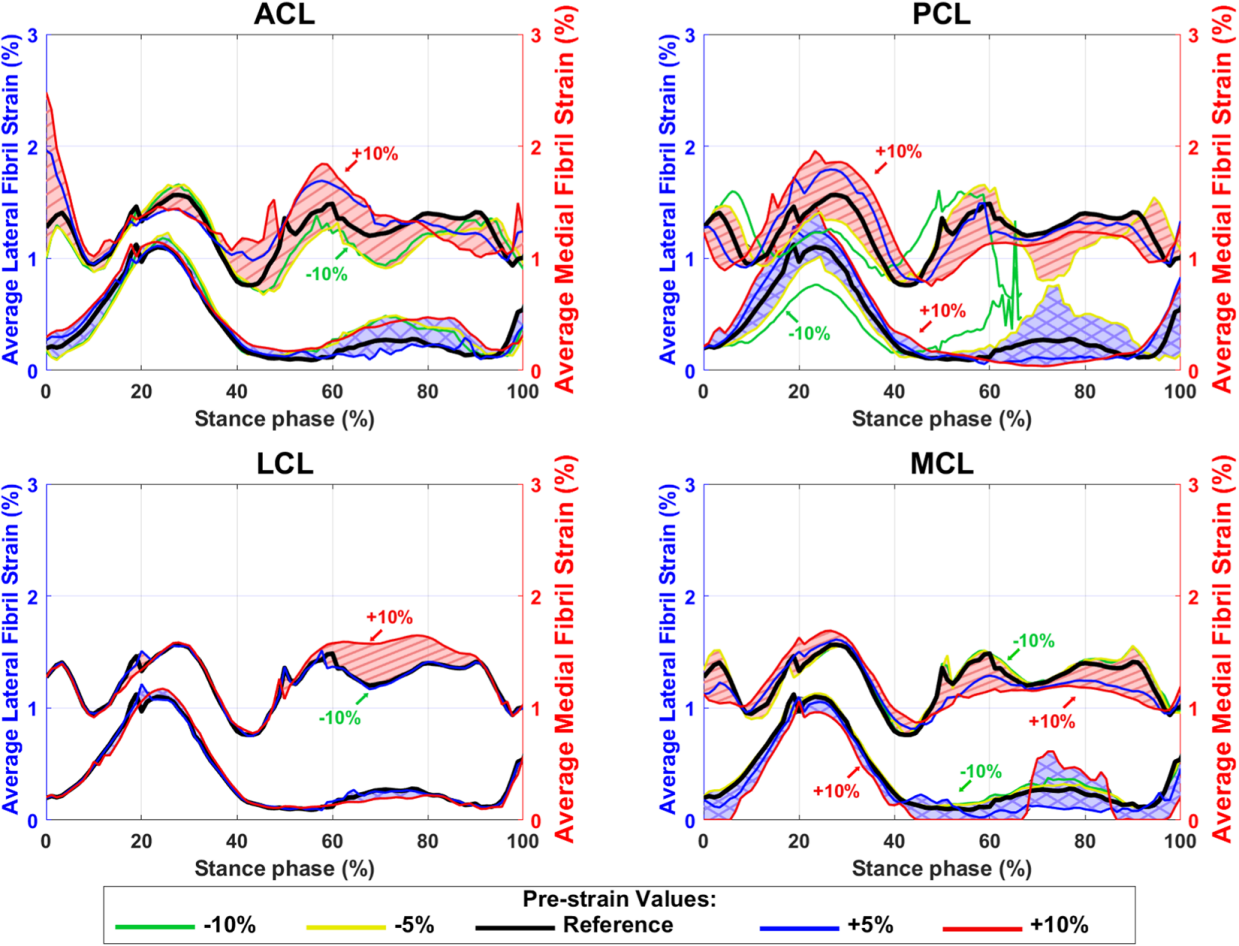
Average tibial cartilage fibril strain within the contact area with different pre-strains of ACL, PCL, LCL, and MCL bundles. The blue shaded areas (with crosslines) show the values on the lateral tibial cartilage (left axis) and the red shaded areas (diagonal lines) show the values on the medial tibial cartilage (right axes).

**Figure 10:**
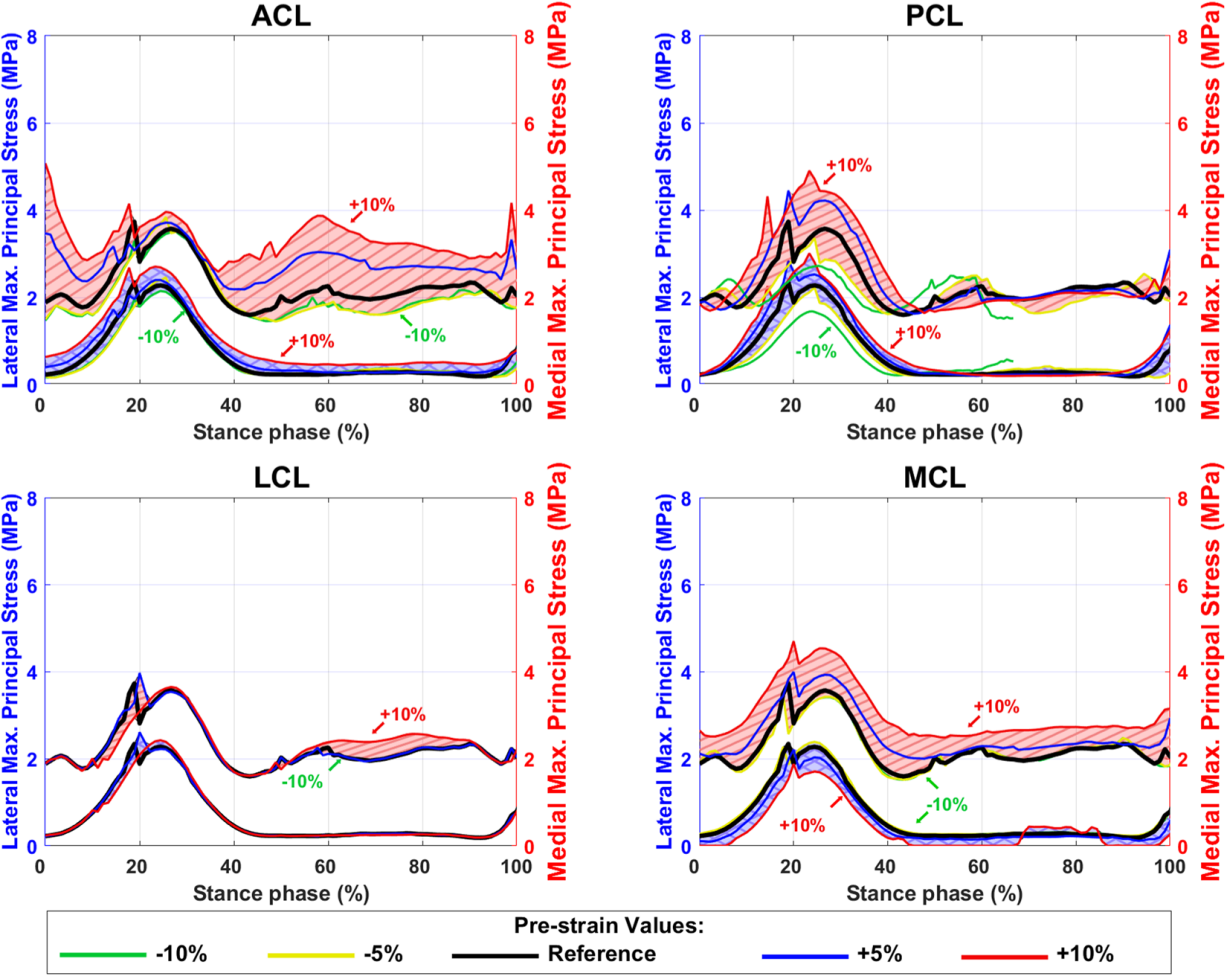
Average maximum principal stress in tibial cartilage inside the contact area with different pre-strains of ACL, PCL, LCL, and MCL bundles. The blue shaded areas (with crosslines) show the values on the lateral tibial cartilage (left axis) and the red shaded area (diagonal lines) show the values on the medial tibial cartilage (right axes).

Figures 9, 10, and supplementary material Fig. S3 show average fibril strain, maximum principal stress and fluid pressure within the tibiofemoral cartilage contact. These are comparable with experimental data as well as previous FE modeling studies^8,14,20,27,41^.

Cartilage responses were altered moderately by increases in ligament pre-strains (Figs. 9, 10, and S3). These alterations were mostly on the medial side rather than on the lateral side. Prior to the midstance, greater pre-strains in PCL and MCL slightly increased the average fibril strain in tibial cartilage, while the pre-strain in ACL and LCL did not affect this parameter considerably (Fig. 9). After the midstance, the average fibril strain was decreased in both medial and lateral compartments when the pre-strain in PCL and MCL increased. In contrast, the average fibril strain was not altered substantially by changing the pre-strain in LCL (Fig. 9).

Maximum principal stress and fluid pressure (Figs. 10 and S3) were slightly increased at the second peak of the GRF by increasing the pre-strain in ACL and MCL, while they were gradually increased at the first peak of the GRF by an increase in the PCL pre-strain. Changing the LCL pre-strain did not alter considerably maximum principal stress and fluid pressure values (Figs. 10 and S3).

## Discussion

The aim of this study was to develop and present a multiscale modeling workflow by combining a subject-specific EMG-assisted MS model with a muscle force driven FE model based on MRIs, EMG signals, and motion data of the subject. The EMG-assisted MS model was used to calculate muscle forces, joint moments and the JCF as well as the knee flexion angle to use as inputs to the FE model. Finally, the FE model utilizing the FRPVE material model was used to calculate secondary kinematics, JCF, contact area, stress, strain (including fibril strain), and fluid pressure of cartilage which all are essential parameters in the evaluation of overload-induced cartilage degeneration^49,93–97^.

In kinematics-driven FE models of the knee joint^20,23,28,98^, translations and rotations (in some cases partly combined with kinetics) of the knee joint were used as inputs. One limitation of this approach is inaccurate estimation of joint secondary kinematics^91,99^ when using gait analysis with skin-attached markers. In addition, it has been shown that the estimated tissue mechanical responses by kinematics-driven FE models are considerably sensitive to the uncertainties in inputs (i.e. secondary kinematics), diametrically opposed to kinetics-driven FE models^44^. Our workflow uses the knee flexion angle as the only kinematic input which has the least probable measurement error among knee joint rotational and translational DOFs^91,100,101^.

Excluding muscle force driven FE models, the external knee joint moments were typically scaled and then applied to the kinetics-driven FE models^11,14,19,27,41,42^. The net joint moments were scaled assuming that muscles generate most of the joint moments. This assumption can alter the joint mechanical loading and the final results^3,43^. In the present study, scaling was not needed since the FE model was driven by muscle forces^18,29^.

Compared to previous muscle force driven FE models, which all used static-optimization methods in the MS models^14,18,23,27–29,32^, the model of the present study utilizes an EMG-assisted approach. Thus, the model of the current study can account for the subject-specific and activity-based muscle activations and co-activation patterns which have been suggested to provide a better estimate of the muscle forces and joint loads^39,71^. These factors may play a crucial role when modeling the knee joint with OA or other MS disorders^12,28,102^, or when simulating rehabilitation exercises.

Muscle forces estimated by the EMG-assisted MS model (Fig. 2C) were in good agreement with previous studies on normal walking^18,74^. The MS model and the FE model with reference ligament pre-strains estimated the maximum JCF of 2.7 and 3.2 times bodyweight, respectively (Fig. 3), which are comparable with *in vivo* measurements and numerical studies^9,40,63,103^. It should be mentioned that in the grand challenge dataset (Fig. 3, the black line) there is a limited effect of the knee joint ligaments and the knee joint surfaces have been made of engineering materials. This may explain higher JCFs produced by our FE model and is also a reason why we could not use that dataset as an input to the models of the current study.

The JCF estimated by the MS model (Fig. 3, red line) was similar to the JCF estimated by the FE model especially when the ligament pre-strain was decreased (Fig. 3, the lower boundary of the shaded area). This small difference resulted from ligaments in the FE model. In fact, the approximately 19% difference in the JCF between the MS model and the FE model (with reference pre-strains of ligaments) is consistent with a previous study^103^ in which Shelburne *et al*. reported that at the time point of the maximum JCF, ligaments carried ~18% of the JCF.

Experimentally measured secondary kinematics of the knee joint^8,91,92^ show considerable variations in both magnitudes and patterns (Fig. 6). However, the estimated rotations and the distal-proximal translation by the FE model were consistent with the experiments from literature (Figs. 6a,b,e). The estimated anteroposterior and mediolateral translations by the FE model had a similar range of motion to those from experiments (Figs. 6c,d). Nonetheless, the variations and discrepancies between the estimated and experimentally measured secondary kinematics could be, e.g., due to different muscle forces, external joint moments, knee orientations, gait style, or ligament pre-strains^29^, which all are subject-specific. For instance, Fig. 6a shows that the internal-external rotation approaches experimentally measured values by the dual fluoroscopy method^92^ when ligament pre-strains are decreased. In addition, the mediolateral component of the total JCF vector (for the analyzed gait trial) was towards the lateral side of the knee joint during the stance phase. This could explain the discrepancy between the estimated mediolateral translation of the femur and the corresponding experimental values from literature (Fig. 6d).

The total tibiofemoral cartilage to cartilage contact area (Fig. 5) was estimated between 250–400 mm^2^ by the FE model, which is comparable with the experimental results (~150 to 600 mm^2^) reported by Gilbert *et al*. ^8^. The average fibril strain during the stance phase (Fig. 9, the black line) was up to 2.7% on the medial and 1.5% on the lateral tibial cartilage, while those were up to 3% and 1.4%, respectively, in previous studies^14,20,27,42^. The average maximum principal stress (Fig. 10, the black line) varied from 1.3 to 4.8 MPa on the medial and from 0.3 to 1.8 MPa on the lateral compartment. This is also consistent with previous studies^14,27,42^ with maximum principal stresses of 1.5–9 MPa and 0–4.5 MPa on the medial and lateral compartments, respectively. The fluid pressure within the tibial cartilage (Fig. S3, the black line) was 1.4–6 MPa on the medial and 0–6 MPa on the lateral joint compartment. Those were 1.5–6 MPa and 0–4 MPa, respectively, in previous computational studies^14,42^.

Both amplitude and mediolateral distribution of the JCF, contact area, and joint kinematics were substantially sensitive to ligament pre-strains. This is consistent with previous studies^104–107^. Figure 4 shows that the JCF amplitude and distribution were mostly affected by ACL and MCL pre-strains while the tibiofemoral cartilage to cartilage contact area was mostly sensitive to the pre-strain of ACL, PCL and MCL (Fig. 5). Joint kinematics were mostly affected by ACL and PCL pre-strains (Figs. 7, 8, and S2).

Increasing ligament pre-strains increased the peak JCF up to 50% and altered the JCF distribution from the medial side to the lateral side and vice versa (Figs. 3 and 4). Therefore, ligament pre-strains could considerably affect modeling results as well as clinical interpretations, for instance, regarding the risk of OA in patients with surgical ACL reconstruction^108,109^.

Despite the high sensitivity of joint kinematics and kinetics to ligament pre-strains, cartilage responses were only moderately altered by changes in ligament pre-strains. This could be explained due to changes in the contact area (Fig. 5) and the nonlinear behavior of the FRPVE material. On the other hand, the fibril strain, the maximum principal stress, and the fluid pressure could increase up to 50% at some time points during the stance phase. However, these increases did not occur at the time points of peak JCF and changes for instance in the absolute values of fibril strain were small (Figs. 9, 10, and S3).

One limitation of this study is modeling the tibiofemoral joint in the MS model as a hinge joint. However, this model has been suggested to be sufficient for estimating muscle forces during walking^6,30,63–68^, consistent with recorded EMGs of the muscles and experimental JCFs^6,30,63–65,67,68^. The estimated abduction-adduction and internal-external moments of this study (Fig. 2d) were in the normal range^110^. However, in activities other than walking, abduction-adduction or internal-external moments of the knee joint could increase significantly^111^, which might void the assumption of modeling the knee joint as a 1 DOF joint. Our future research aims to consider secondary kinematics in the MS model and further to evaluate the influence of knee joint DOFs on tissue mechanics.

The other limitation of the study was the lack of experimental data to compare the ligament pre-strain effects on cartilage stress, strain, and fluid pressure results. However, our results are comparable with previous FE models^8,14,27,41^. Different fiber bundles of each ligament could not be distinguished from MRIs. Thus, the average pre-strain of fiber bundles was assigned to the corresponding ligaments. This is a limitation of the study, since each ligament fiber bundle might have different pre-strain^112^, fiber direction, and different mechanical properties. Nonetheless, if each ligament could be separated in different bundles, stresses and strains within single ligaments would change, but the overall effect on the knee joint mechanics should remain small. The use of only one participant was also a limitation which may not represent kinematics and kinetics of different subjects. However, secondary kinematics calculated by the FE model were consistent with the results from literature (Fig. 6)^8,91,92^. Moreover, the main aim of the study was to introduce a workflow that can be utilized in further studies rather than presenting typical values for the joint and cartilage mechanics.

The presented workflow shows potential to estimate movements of the femur and patella during different activities or muscle activations that may be of particular relevance to the onset and progression of KOA^93^. Various invasive surgeries (such as osteotomy, ligament reconstruction, and tendon transfer surgery) or non-invasive methods for gait modification (such as orthosis and lateral wedges) could be simulated and analyzed with our modeling approach. In particular, contribution of muscles through EMG to the cartilage responses, and modification of muscle activities, e.g. for rehabilitation purposes, can be simulated by our model.

From the tissue level point of view, early changes in KOA include several factors such as PG depletion and loss of cartilage surface integrity together with collagen fibrillation and formation of cartilage fissures. Based on earlier studies, stresses and strains (including deviatoric, shear and maximum principal strains) in the cartilage and/or in collagen fibrils, as well as fluid flow within the cartilage, may be used to predict tissue failure, cartilage degeneration, and progression of KOA^94–97,113^. The current model, now with the EMG-assisted muscle force driven approach and the FRPVE material model, could be applied for different patient groups in the prediction of collagen damage and PG loss during the progression of OA and evaluation of the effect of interventions.

Time is important when applying any method for clinical purposes. In the presented workflow, it takes about a month to segment and mesh tissues from MRIs and to create and simulate successfully the EMG-assisted MS model linked with the present FE model. Nonetheless, we are extensively working on our atlas-based modeling approach to be able to create a FE model in minutes^114^. The atlas-based FE model is not yet muscle force driven and is limited to the tibiofemoral contact and subjects with healthy cartilage^114^.

In conclusion, we presented a novel workflow for multi-scale modeling of the knee joint. The EMG-assisted MS model linked with the muscle force driven FE model with FRP(V)E cartilages and menisci can be used for evaluating knee joint kinematics, load distribution and tissue responses in different activities where muscle force alterations are important. According to the sensitivity analysis, we conclude that ligament pre-strains should be implemented with great accuracy when analyzing kinematics and kinetics of the knee joint (especially for pre-strain values higher than the reference values). Nonetheless, if the modeling focuses on tissue responses (such as fibril strain, stress, and fluid pressure within the cartilage), small alterations in ligament pre-strains will still lead to acceptable results.

## Supplementary information

Supplementary Material

## References

[1] Ruiz D (2013). The Direct and Indirect Costs to Society of Treatment for End-Stage Knee Osteoarthritis. J. Bone Jt. Surgery-American.

[2] Fisher NM, Pendergast DR, Gresham GE, Calkins E (1991). Muscle rehabilitation: Its effect on muscular and functional performance of patients with knee osteoarthritis. Arch. Phys. Med. Rehabil..

[3] Sharma L (1998). Knee adduction moment, serum hyaluronan level, and disease severity in medial tibiofemoral osteoarthritis. Arthritis Rheum..

[4] Baliunas AJ (2002). Increased knee joint loads during walking are present in subjects with knee osteoarthritis. Osteoarthr. Cartil..

[5] Schnitzer TJ, Popovich JM, Andersson GBJ, Andriacchi TP (1993). Effect of piroxicam on gait in patients with osteoarthritis of the knee. Arthritis Rheum..

[6] DeMers MS, Pal S, Delp SL (2014). Changes in tibiofemoral forces due to variations in muscle activity during walking. J. Orthop. Res..

[7] Wang H (2014). Image based weighted center of proximity versus directly measured knee contact location during simulated gait. J. Biomech..

[8] Gilbert S (2014). Dynamic contact mechanics on the tibial plateau of the human knee during activities of daily living. J. Biomech..

[9] Fregly BJ (2012). Grand challenge competition to predict *in vivo* knee loads. J. Orthop. Res..

[10] Caruntu, D. I. Knee Joint Modeling. in *Volume 1: 21st Biennial Conference on Mechanical Vibration and Noise, Parts A, B, and C* 673–678, 10.1115/DETC2007-35029 (ASME, 2007).

[11] Liukkonen, M. K. *et al*. Evaluation of the Effect of Bariatric Surgery-Induced Weight Loss on Knee Gait and Cartilage Degeneration. *J. Biomech. Eng*. **140** (2018). 10.1115/1.4038330 29101403

[12] Lloyd DG, Besier TF (2003). An EMG-driven musculoskeletal model to estimate muscle forces and knee joint moments *in vivo*. J. Biomech..

[13] Fernandez, J. *et al*. Multiscale musculoskeletal modelling, data–model fusion and electromyography-informed modelling. *Interface Focus* **6** (2016). 10.1098/rsfs.2015.0084 PMC4759749 27051510

[14] Halonen KS (2017). Workflow assessing the effect of gait alterations on stresses in the medial tibial cartilage - Combined musculoskeletal modelling and finite element analysis. Sci. Rep..

[15] Mesfar W, Shirazi-Adl A (2005). Biomechanics of the knee joint in flexion under various quadriceps forces. Knee.

[16] Arnold EM, Ward SR, Lieber RL, Delp SL (2010). A Model of the Lower Limb for Analysis of Human Movement. Ann. Biomed. Eng..

[17] Venäläinen MS (2016). Quantitative Evaluation of the Mechanical Risks Caused by Focal Cartilage Defects in the Knee. Sci. Rep..

[18] Navacchia A, Hume DR, Rullkoetter PJ, Shelburne KB (2019). A computationally efficient strategy to estimate muscle forces in a finite element musculoskeletal model of the lower limb. J. Biomech..

[19] Tanska P, Mononen ME, Korhonen RK (2015). A multi-scale finite element model for investigation of chondrocyte mechanics in normal and medial meniscectomy human knee joint during walking. J. Biomech..

[20] Mononen ME, Jurvelin JS, Korhonen RK (2015). Implementation of a gait cycle loading into healthy and meniscectomised knee joint models with fibril-reinforced articular cartilage. Comput. Methods Biomech. Biomed. Engin..

[21] Räsänen LP (2013). Implementation of subject-specific collagen architecture of cartilage into a 2D computational model of a knee joint-data from the osteoarthritis initiative (OAI). J. Orthop. Res..

[22] Skipper Andersen M, de Zee M, Damsgaard M, Nolte D, Rasmussen J (2017). Introduction to Force-Dependent Kinematics: Theory and Application to Mandible Modeling. J. Biomech. Eng..

[23] Adouni M, Shirazi-Adl A, Shirazi R (2012). Computational biodynamics of human knee joint in gait: From muscle forces to cartilage stresses. J. Biomech..

[24] Marouane H, Shirazi-Adl A, Adouni M (2016). Alterations in knee contact forces and centers in stance phase of gait: A detailed lower extremity musculoskeletal model. J. Biomech..

[25] Meireles S (2016). Knee contact forces are not altered in early knee osteoarthritis. Gait Posture.

[26] Pizzolato C (2015). CEINMS: A toolbox to investigate the influence of different neural control solutions on the prediction of muscle excitation and joint moments during dynamic motor tasks. J. Biomech..

[27] Kłodowski A (2016). Merge of motion analysis, multibody dynamics and finite element method for the subject-specific analysis of cartilage loading patterns during gait: differences between rotation and moment-driven models of human knee joint. Multibody Syst. Dyn..

[28] Adouni M, Shirazi-Adl A (2014). Evaluation of knee joint muscle forces and tissue stresses-strains during gait in severe OA versus normal subjects. J. Orthop. Res..

[29] Lenhart RL, Kaiser J, Smith CR, Thelen DG (2015). Prediction and validation of load-dependent behavior of the tibiofemoral and patellofemoral joints during movement. Ann. Biomed. Eng..

[30] Marra MA (2015). A subject-specific musculoskeletal modeling framework to predict *in vivo* mechanics of total knee arthroplasty. J. Biomech. Eng..

[31] Manal K, Buchanan TS (2013). An Electromyogram-Driven Musculoskeletal Model of the Knee to Predict *in vivo* Joint Contact Forces During Normal and Novel Gait Patterns. J. Biomech. Eng..

[32] Marouane H, Shirazi-Adl A, Adouni M (2017). 3D active-passive response of human knee joint in gait is markedly altered when simulated as a planar 2D joint. Biomech. Model. Mechanobiol..

[33] Heiden TL, Lloyd DG, Ackland TR (2009). Knee joint kinematics, kinetics and muscle co-contraction in knee osteoarthritis patient gait. Clin. Biomech..

[34] Hubley-Kozey CL, Hill NA, Rutherford DJ, Dunbar MJ, Stanish WD (2009). Co-activation differences in lower limb muscles between asymptomatic controls and those with varying degrees of knee osteoarthritis during walking. Clin. Biomech..

[35] Schmitt LC, Rudolph KS (2008). Muscle stabilization strategies in people with medial knee osteoarthritis: The effect of instability. J. Orthop. Res..

[36] Nikooyan AA (2012). An EMG-driven musculoskeletal model of the shoulder. Hum. Mov. Sci..

[37] Cholewicki, J., McGill, S. M. & Norman, R. W. Comparison of muscle forces and joint load from an optimization and EMG assisted lumbar spine model: Towards development of a hybrid approach. *J. Biomech*. **28** (1995). 10.1016/0021-9290(94)00065-c 7730390

[38] Falisse A, Van Rossom S, Jonkers I, De Groote F (2017). EMG-Driven Optimal Estimation of Subject-SPECIFIC Hill Model Muscle-Tendon Parameters of the Knee Joint Actuators. IEEE Trans. Biomed. Eng..

[39] Wesseling M (2015). Muscle optimization techniques impact the magnitude of calculated hip joint contact forces. J. Orthop. Res..

[40] Hoang HX, Diamond LE, Lloyd DG, Pizzolato C (2019). A calibrated EMG-informed neuromusculoskeletal model can appropriately account for muscle co-contraction in the estimation of hip joint contact forces in people with hip osteoarthritis. J. Biomech..

[41] Halonen KS (2016). Importance of Patella, Quadriceps Forces, and Depthwise Cartilage Structure on Knee Joint Motion and Cartilage Response During Gait. J. Biomech. Eng..

[42] Orozco GA, Tanska P, Mononen ME, Halonen KS, Korhonen RK (2018). The effect of constitutive representations and structural constituents of ligaments on knee joint mechanics. Sci. Rep..

[43] Astephen JL, Deluzio KJ, Caldwell GE, Dunbar MJ (2008). Biomechanical changes at the hip, knee, and ankle joints during gait are associated with knee osteoarthritis severity. J. Orthop. Res..

[44] Adouni M, Shirazi-Adl A (2014). Partitioning of knee joint internal forces in gait is dictated by the knee adduction angle and not by the knee adduction moment. J. Biomech..

[45] Ihn JC, Kim SJ, Park IH (1993). *In vitro* study of contact area and pressure distribution in the human knee after partial and total meniscectomy. Int. Orthop..

[46] Radin, E. L., de Lamotte, F. & Maquet, P. Role of the menisci in the distribution of stress in the knee. *Clin. Orthop. Relat. Res*. 290–4 (1984). 6546709

[47] Julkunen P, Harjula T, Marjanen J, Helminen HJ, Jurvelin JS (2009). Comparison of single-phase isotropic elastic and fibril-reinforced poroelastic models for indentation of rabbit articular cartilage. J. Biomech..

[48] Mukherjee N, Wayne JS (1998). Load sharing between solid and fluid phases in articular cartilage: II — comparison of experimental results and u-p finite element predictions. J. Biomech. Eng..

[49] Mäkelä JT, Han S-K, Herzog W, Korhonen R (2015). Very early osteoarthritis changes sensitively fluid flow properties of articular cartilage. J. Biomech..

[50] Li LP, Gu KB (2011). Reconsideration on the use of elastic models to predict the instantaneous load response of the knee joint. Proc. Inst. Mech. Eng. Part H J. Eng. Med..

[51] Wilson W, van Donkelaar CC, van Rietbergen B, Huiskes R (2005). A fibril-reinforced poroviscoelastic swelling model for articular cartilage. J. Biomech..

[52] Dhaher YY, Kwon T-H, Barry M (2010). The effect of connective tissue material uncertainties on knee joint mechanics under isolated loading conditions. J. Biomech..

[53] Shelburne KB, Torry MR, Pandy MG (2006). Contributions of muscles, ligaments, and the ground-reaction force to tibiofemoral joint loading during normal gait. J. Orthop. Res..

[54] Carey, R. E., Zheng, L., Aiyangar, A. K., Harner, C. D. & Zhang, X. Subject-specific finite element modeling of the tibiofemoral joint based on ct, magnetic resonance imaging and dynamic stereo-radiography data *in vivo*. *J. Biomech. Eng*. **136** (2014). 10.1115/1.4026228 PMC4023807 24337180

[55] Liu F (2010). *In vivo* tibiofemoral cartilage deformation during the stance phase of gait. J. Biomech..

[56] Räsänen LP (2016). Three dimensional patient-specific collagen architecture modulates cartilage responses in the knee joint during gait. Comput. Methods Biomech. Biomed. Engin..

[57] Räsänen, L. P. *et al*. Spatial variation of fixed charge density in knee joint cartilage from sodium MRI – Implication on knee joint mechanics under static loading. *J. Biomech*., 10.1016/j.jbiomech.2016.09.011 (2016). 10.1016/j.jbiomech.2016.09.011 27667478

[58] Kang, K. T., Kim, S. H., Son, J., Lee, Y. H. & Chun, H. J. Computational model-based probabilistic analysis of *in vivo* material properties for ligament stiffness using the laxity test and computed tomography. *J. Mater. Sci. Mater. Med*. **27** (2016). 10.1007/s10856-016-5797-z 27787809

[59] Li G, Suggs J, Gill T (2002). The Effect of Anterior Cruciate Ligament Injury on Knee Joint Function under a Simulated Muscle Load: A Three-Dimensional Computational Simulation. Ann. Biomed. Eng..

[60] Smith CR, Lenhart RL, Kaiser J, Vignos MF, Thelen DG (2016). Influence of Ligament Properties on Tibiofemoral Mechanics in Walking. J. Knee Surg..

[61] Baldwin MA (2012). Dynamic finite element knee simulation for evaluation of knee replacement mechanics. J. Biomech..

[62] Delp, S. L. *et al*. *OpenSim: Open-Source Software to Create and Analyze Dynamic Simulations of Movement*. *Biomedical Engineering, IEEE Transactions on* **54** (2007). 10.1109/TBME.2007.901024 18018689

[63] Gerus P (2013). Subject-specific knee joint geometry improves predictions of medial tibiofemoral contact forces. J. Biomech..

[64] Yamaguchi GT, Zajac FE (1989). A planar model of the knee joint to characterize the knee extensor mechanism. J. Biomech..

[65] Moeinzadeh MH, Engin AE, Akkas N (1983). Two-dimensional dynamic modelling of human knee joint. J. Biomech..

[66] Wongchaisuwat C, Hemami H, Hines MJ (1984). Control exerted by ligaments. J. Biomech..

[67] Nisell R, Németh G, Ohlsén H (1986). Joint forces in extension of the knee: Analysis of a mechanical model. Acta Orthop. Scand..

[68] Nisell R (1985). Mechanics of the knee. A study of joint and muscle load with clinical applications. Acta Orthop. Scand. Suppl..

[69] Thelen DG, Anderson FC (2006). Using computed muscle control to generate forward dynamic simulations of human walking from experimental data. J. Biomech..

[70] Thelen DG, Anderson FC, Delp SL (2003). Generating dynamic simulations of movement using computed muscle control. J. Biomech..

[71] Ramsay, J. W., Buchanan, T. S. & Higginson, J. S. EMG-driven Muscle Activations Tune Post-Stroke Computed Muscle Control Simulations. in *American Society of Biomechanics» 2011 Annual Meeting* 657–658 (2011).

[72] van Arkel RJ, Modenese L, Phillips ATM, Jeffers JRT (2013). Hip abduction can prevent posterior edge loading of hip replacements. J. Orthop. Res..

[73] Higginson JS, Ramsay JW, Buchanan TS (2012). Hybrid models of the neuromusculoskeletal system improve subject-specificity. Proc. Inst. Mech. Eng. H..

[74] Anderson FC, Pandy MG (2001). Dynamic Optimization of Human Walking. J. Biomech. Eng..

[75] Julkunen P, Kiviranta P, Wilson W, Jurvelin JS, Korhonen RK (2007). Characterization of articular cartilage by combining microscopic analysis with a fibril-reinforced finite-element model. J. Biomech..

[76] Wilson W, van Donkelaar CC, van Rietbergen B, Ito K, Huiskes R (2004). Stresses in the local collagen network of articular cartilage: a poroviscoelastic fibril-reinforced finite element study. J. Biomech..

[77] Dabiri Y, Li LP (2013). Influences of the depth-dependent material inhomogeneity of articular cartilage on the fluid pressurization in the human knee. Med. Eng. Phys..

[78] Makris EA, Hadidi P, Athanasiou KA (2011). The knee meniscus: Structure–function, pathophysiology, current repair techniques, and prospects for regeneration. Biomaterials.

[79] Böttcher P, Zeissler M, Maierl J, Grevel V, Oechtering G (2009). Mapping of split-line pattern and cartilage thickness of selected donor and recipient sites for autologous osteochondral transplantation in the canine stifle joint. Vet. Surg..

[80] Leo BM, Turner MA, Diduch DR (2004). Split-line pattern and histologic analysis of a human osteochondral plug graft. Arthrosc. J. Arthrosc. Relat. Surg..

[81] Goodwin DW (2004). Macroscopic Structure of Articular Cartilage of the Tibial Plateau: Influence of a Characteristic Matrix Architecture on MRI Appearance. Am. J. Roentgenol..

[82] Benninghoff, A. Form und Bau der Gelenkknorpel in ihren Beziehungen zur Funktion. *Zeitschrift für Zellforschung und Mikroskopische Anatomie* **2**(5), 783–862 (1925).

[83] Below S, Arnoczky SP, Dodds J, Kooima C, Walter N (2002). The split-line pattern of the distal femur: A consideration in the orientation of autologous cartilage grafts. Arthrosc. J. Arthrosc. Relat. Surg..

[84] Blankevoort L, Huiskes R (1991). Ligament-bone interaction in a three-dimensional model of the knee. J. Biomech. Eng..

[85] Blankevoort, L., Huiskes, R. & de Lange, A. The envelope of passive knee joint motion. *J. Biomech*. **21** (1988). 10.1016/0021-9290(88)90280-1 3182875

[86] Butler DL, Kay MD, Stouffer DC (1986). Comparison of material properties in fascicle-bone units from human patellar tendon and knee ligaments. J. Biomech..

[87] Villegas DF, Maes JA, Magee SD, Haut Donahue TL (2007). Failure properties and strain distribution analysis of meniscal attachments. J. Biomech..

[88] Naghibi Beidokhti H (2017). The influence of ligament modelling strategies on the predictive capability of finite element models of the human knee joint. J. Biomech..

[89] Mesfar W, Shirazi-Adl A (2006). Biomechanics of changes in ACL and PCL material properties or prestrains in flexion under muscle force-implications in ligament reconstruction. Comput. Methods Biomech. Biomed. Engin..

[90] Halonen KS (2016). Optimal graft stiffness and pre-strain restore normal joint motion and cartilage responses in ACL reconstructed knee. J. Biomech..

[91] Benoit DL (2006). Effect of skin movement artifact on knee kinematics during gait and cutting motions measured *in vivo*. Gait Posture.

[92] Kozanek M (2009). Tibiofemoral kinematics and condylar motion during the stance phase of gait. J. Biomech..

[93] Andriacchi TP (2004). A Framework for the *in vivo* Pathomechanics of Osteoarthritis at the Knee. Ann. Biomed. Eng..

[94] Hosseini SM, Wilson W, Ito K, van Donkelaar CC (2014). A numerical model to study mechanically induced initiation and progression of damage in articular cartilage. Osteoarthr. Cartil..

[95] Wilson W (2006). Causes of mechanically induced collagen damage in articular cartilage. J. Orthop. Res..

[96] Liukkonen MK (2017). Simulation of Subject-Specific Progression of Knee Osteoarthritis and Comparison to Experimental Follow-up Data: Data from the Osteoarthritis Initiative. Sci. Rep..

[97] Mononen ME, Tanska P, Isaksson H, Korhonen RK (2016). A novel method to simulate the progression of collagen degeneration of cartilage in the knee: Data from the osteoarthritis initiative. Sci. Rep..

[98] Hopkins AR, New AM, Rodriguez-y-Baena F, Taylor M (2010). Finite element analysis of unicompartmental knee arthroplasty. Med. Eng. Phys..

[99] Reinschmidt, C., Van Den Bogert, A. J., Nigg, B. M., Lundberg, A. & Murphy, N. Effect of skin movement on the analysis of skeletal knee joint motion during running. *J. Biomech*., 10.1016/S0021-9290(97)00001-8 (1997). 10.1016/s0021-9290(97)00001-8 9239553

[100] Myers CA, Laz PJ, Shelburne KB, Davidson BS (2015). A Probabilistic Approach to Quantify the Impact of Uncertainty Propagation in Musculoskeletal Simulations. Ann. Biomed. Eng..

[101] Cappozzo A, Catani F, Leardini A, Benedetti M, Della Croce U (1996). Position and orientation in space of bones during movement: experimental artefacts. Clin. Biomech..

[102] Killen BA (2018). Individual muscle contributions to tibiofemoral compressive articular loading during walking, running and sidestepping. J. Biomech..

[103] Shelburne KB, Torry MR, Pandy MG (2005). Muscle, Ligament, and Joint-Contact Forces at the Knee during Walking. Med. Sci. Sport. Exerc.

[104] Crottet D (2007). Ligament balancing in TKA: Evaluation of a force-sensing device and the influence of patellar eversion and ligament release. J. Biomech..

[105] Smith CR, Vignos MF, Lenhart RL, Kaiser J, Thelen DG (2016). The Influence of Component Alignment and Ligament Properties on Tibiofemoral Contact Forces in Total Knee Replacement. J. Biomech. Eng..

[106] Fukubayashi, T., Torzilli, P. A., Sherman, M. F. & Warren, R. *An in vitro biomechanical evaluation of anterior-posterior motion of the knee*. *Tibial displacement, rotation, and torque*. *The Journal of bone and joint surgery. American volume* **64** (1982). 7056781

[107] Melby A, Noble JS, Askew MJ, Boom AA, Hurst FW (1991). The effects of graft tensioning on the laxity and kinematics of the anterior cruciate ligament reconstructed knee. Arthrosc. J. Arthrosc. Relat. Surg..

[108] Butler RJ, Minick KI, Ferber R, Underwood F (2009). Gait mechanics after ACL reconstruction: implications for the early onset of knee osteoarthritis. Br. J. Sports Med..

[109] Barenius B (2014). Increased Risk of Osteoarthritis After Anterior Cruciate Ligament Reconstruction. Am. J. Sports Med..

[110] Manal K, McClay I, Richards J, Galinat B, Stanhope S (2002). Knee moment profiles during walking: Errors due to soft tissue movement of the shank and the influence of the reference coordinate system. Gait Posture.

[111] Shirazi-Adl A (1989). On the fibre composite material models of disc annulus—Comparison of predicted stresses. J. Biomech..

[112] Harris, M. D. *et al*. A Combined Experimental and Computational Approach to Subject-Specific Analysis of Knee Joint Laxity. *J. Biomech. Eng*. **138** (2016). 10.1115/1.4033882 PMC4967880 27306137

[113] Danso EK, Honkanen JTJ, Saarakkala S, Korhonen RK (2014). Comparison of nonlinear mechanical properties of bovine articular cartilage and meniscus. J. Biomech..

[114] Mononen ME, Liukkonen MK, Korhonen RK (2018). Utilizing Atlas-Based Modeling to Predict Knee Joint Cartilage Degeneration: Data from the Osteoarthritis Initiative. Ann. Biomed. Eng..

