# Supplementary material for: EMG-Assisted Muscle Force Driven Finite Element Model of the Knee Joint with Fibril-Reinforced Poroelastic Cartilages and Menisci
Source: Sci Rep. 2020 Feb 20;10:3026. doi: 10.1038/s41598-020-59602-2 (PMC7033219; doi:10.1038/s41598-020-59602-2)
Supplement: Supplementary file 1 — Supplementary Material [file 41598_2020_59602_MOESM1_ESM.pdf]

Supplementary material

EMG-Assisted Muscle Force Driven Finite Element Model of the Knee Joint with Fibril-Reinforced Poroelastic Cartilages and Menisci

A. Esrafilian[1], L. Stenroth[1], M.E. Mononen[1], P. Tanska[1], J. Avela[2], R.K. Korhonen[1]

[1] Department of Applied Physics, University of Eastern Finland, Kuopio, Finland

[2] NeuroMuscular Research Center, Unit of Biology of Physical Activity, Faculty of Sport and Health Sciences, University of Jyväskylä, Jyväskylä, Finland

Introduction

Table S1 includes a literature review on the most relevant previously developed MS linked with FE models. A short description and limitations of those studies has been mentioned.

Methods

1. Gait data and MS model

Fig. 1 (see the manuscript) illustrates the workflow of this study. One healthy subject (male, 33 years old, 78 kg, 1.77 m) participated in this study for experimental data collection. The participant signed a written consent. Walking trials with the preferred speed of the subject were conducted at the gait analysis laboratory of the Faculty of Sport and Health Sciences, University of Jyväskylä. Marker trajectories (120 Hz, MX system, Vicon, UK), ground reaction forces (GRF, 1200 Hz, two force plates, OR6-6, AMTI, USA), and EMG signals (1200 Hz, Telemyo 2400T-G2, Noraxon, USA) were recorded from the trials. EMG signals were measured from vastus lateralis, rectus femoris, long head of biceps femoris, semitendinousus, medial gastrocnemius, soleus, and gluteus maximus during walking. The skin was shaved, lightly rubbed with sandpaper and cleaned with alcohol, and bipolar surface electrodes were located based on European recommendation for surface EMG [1].

Table S1. A literature review on the most relevant previously developed MS models linked with FE models

Reference	Description of the knee joint in the models		Limitations	
	The MS model	The FE model	The MS model	The FE model
Halonen et al., 2017 [2]	12 DOFs knee joint.	- Cartilages are modeled as fibril-reinforced poroviscoelastic<br>- Menisci are modeled as fibril-reinforced poroelastic	*- The muscle force estimation is not EMG-assisted*	*- The FE model is not muscle force driven*
Liukkonen et al., 2018 [3]	1 DOF knee joint	- Cartilages are modeled as fibril-reinforced poroviscoelastic<br>- Menisci are modeled as fibril-reinforced poroelastic	*- The muscle force estimation is not EMG-assisted*	*- The FE model is not muscle force driven*
Klodowski et al., 2015 [4]	1 DOF knee joint	- Cartilages are modeled as fibril-reinforced poroviscoelastic<br>- Menisci are modeled as fibril-reinforced poroelastic material	*- The muscle force estimation is not EMG-assisted*	*- The FE model is not muscle force driven*
Marouane et al., 2017 [5]<br>Adouni et al., 2014 [6]<br>Adouni et al. 2012 [7]	3 DOFs knee joint	- Cartilages are modeled as fibril-reinforced hyperelastic<br>- Menisci are modeled as fibril-reinforced nonhomogeneous composite	*- The muscle force estimation is not EMG-assisted*<br>*- The secondary kinematics are needed since the varus-valgus and internal-external rotation of the femur are constrained to the secondary kinematics.*	*- The secondary kinematics are needed since the varus-valgus and internal-external rotation of the femur are constrained to the secondary kinematics*<br>*- Soft tissues neither consider fluid-flow independent (collagen viscoelasticity) nor fluid flow dependent (poroelasticity) viscoelasticities*
Lenhart et al., 2015 [8]	12 DOFs knee joint.	- An elastic foundation model	*- The muscle force estimation is not EMG-assisted*	*- Cartilages are modeled as nonlinear elastic*<br>*- Menisci are not included*
Navacchia et al., 2019 [9]	1 DOF knee joint	- Nonlinear elastic cartilages	*- The muscle force estimation is not EMG-assisted*	*- Cartilages are modeled as nonlinear elastic*<br>*- Menisci are not included*
Current Study	1 DOF MS knee joint model, 12 DOFs muscle force driven FE knee joint model, fibril-reinforced poroviscoelastic cartilages, fibril-reinforced poroelastic menisci, analysis of a continuous gait cycle			

EMG signals were bandpass filtered between 10 and 500 Hz and notch filtered (50 Hz) to remove the power line noise. The MOtoNMS toolbox was used to analyze and obtain the enveloped EMG signals [10]. Maximum isometric contraction of the muscles was performed against a resistance to acquire maximal muscle activities of the muscles for normalization of the muscle activities measured during walking. For each muscle group, three maximal trials were performed. In our approach, the MS model was used to estimate muscle forces (while secondary kinematics were estimated by the FE model). Therefore, one degree of freedom (DOF), sliding hinge type knee joint model was deemed sufficient for estimating muscle forces and resultant JCFs [11]. Thus, a standard Gait2392 MS model of the OpenSim (v.3.3,SimTK) software was selected [12]. The geometry, mass and inertial properties, as well as muscle properties which depends on length (such as optimal fiber length and tendon slack length) of the MS model, were scaled based on the static trial of the subject. Then the residual reduction algorithm (RRA) toolbox was used to make joint angles and body translations more dynamically consistent with the measured grand reaction forces (GRF) and moments [12].

The EMG-assisted Computed Muscle Control (CMC) toolbox of the OpenSim software (with its default activation dynamics and force-excitation relations of the muscles) was used to estimate muscle forces along with their direction of action as well as effective moment arms [13,14]. The toolbox uses an optimization technique as well as a closed-loop proportional-integral-derivative (PID) controller to estimate muscle forces while tracking the measured gait kinematics. As a result, each muscle excitation can vary from 0.02 (considered as zero excitation) to 1 (fully excited) without any penalization factor [13]. Nonetheless, in an EMG-assisted MS model, a penalty (or weight) factor forces the optimization algorithm to find each muscle excitation within a range of the measured EMG of the corresponding muscle. And for those muscles without measured EMGs, any excitation level within the default range (0.02-1) is considered as an acceptable solution. In other words, the muscle activations were found by: 1) minimizing the error between the external moment on the knee joint and the moment generated by muscles, 2) minimizing the estimated muscle activations, and 3) estimating the activation of the measured muscles within a specific range of the measured EMGs.

Thus, we calculated normalized muscle activation levels from the EMG signal of the measured muscles and imported them into the CMC toolbox (more information on EMG measurements and analysis is presented in the supplementary material). As a penalty factor, only the solution within ±10% variation from measured EMGs was accepted for the muscles (consequently, enveloped EMG signals and corresponding muscle forces presented in Fig. 2C

have the same patterns). The acceptable excitation range for the rest of the muscles were set to the default values of the CMC toolbox. In summary, the MS model was used to calculate the knee joint flexion angle (inverse kinematics) and the knee joint moments (inverse dynamics), and then to estimate muscle forces (CMC toolbox) and the JCF (joint reaction analysis in OpenSim) as inputs to the FE model (Fig. 2).

2. FE model

2.1. Geometry

The FE model geometry including femoral, tibial and patellar cartilage, and menisci was manually segmented from the subject's MR images [15] in MIMICS v.15.01 (Materialise, Leuven, Belgium). A custom-made Matlab script (Mathworks, Natick, MA, USA) converted segmented surfaces to solid geometries and finally, all solid parts were imported into Abaqus FE software (Abaqus v6.142, Dassault Systèmes, RI, USA). 8-node hexahedral porous elements (C3D8P) were used to mesh cartilages and menisci. All the bones were modeled as rigid to reduce computational costs.

2.2. Material properties of cartilages, menisci, and knee ligaments

2.2.1. Material properties of cartilages and menisci

Table S2 shows the material parameters for the knee joint cartilages and menisci. Cartilages were modeled as a FRPVE material [16,17] and menisci as a FRPE material [16,18,19]. Moreover, the depth-dependent Benninghoff-type arcade architecture of collagen fibrils with split-lines was implemented for femoral, tibial, and patellar cartilages [20–23]. More details on implementation can be found from our previous studies [15,24].

The total stress in the cartilage and menisci ($\boldsymbol{\sigma}_t$) consists of the non-fibrillar matrix stress ($\boldsymbol{\sigma}_{nf}$), collagen fibril stress ($\boldsymbol{\sigma}_f$), and fluid pressure (p):

$$\boldsymbol{\sigma}_t = \boldsymbol{\sigma}_{nf} + \boldsymbol{\sigma}_f - p\mathbf{I} \quad \text{(S1)}$$

where **I** is the unit tensor. The non-fibrillar matrix in cartilages and menisci was modeled by compressible neo-Hookean properties. The stress within the non-fibrillar matrix is given by [25]:

$$\boldsymbol{\sigma}_{nf} = K\frac{\ln(J)}{J}\mathbf{I} + \frac{G}{J}(\mathbf{F}\mathbf{F}^T - J^{2/3}\mathbf{I}) \quad \text{(S2)}$$

$$K = \frac{E_m}{3(1-2\nu_m)} \quad \text{(S3)}$$

$$G = \frac{E_m}{2(1+\nu_m)} \tag{S4}$$

where K and G are the bulk and shear moduli of the non-fibrillar matrix, J is the determinant of the deformation tensor **F**, E_m is the Young's modulus of the nonfibrillar matrix, and ν_m is the Poisson's ratio of the nonfibrillar matrix. A strain dependent permeability k [26] is given by [27]:

$$k = k_0 \left(\frac{1+e}{1+e_0}\right)^M \tag{S5}$$

where k_0 is the initial permeability, e is the current and e_0 the initial void ratio, and M is a positive constant. The fluid fraction was assumed to be depth-dependent in equilibrium [28] as:

$$n_{f,eq} = 0.85 - 0.15 d_n \tag{S6}$$

where d_n is the normalized depth (0 at the surface and 1 at the cartilage-bone interface).

The collagen fibrils of cartilage were modeled as a viscoelastic material. In the material model, a non-linear spring (with the strain-dependent modulus $E_\varepsilon \varepsilon_f$) is in series with a linear dashpot (with the damping coefficient η). This nonlinear spring-dashpot system is in parallel with a linear spring (with the initial modulus E_0). The collagen fibrils within the menisci were modeled as a strain-dependent elastic material by a linear spring in parallel with a non-linear spring (with E_0 and $E_\varepsilon \varepsilon_f$). Fibrils were assumed to resist only tension, thus the collagen fibril stress of the cartilage was formulated as [29]:

$$\boldsymbol{\sigma}_f = \begin{cases} -\frac{\eta}{2\sqrt{(\boldsymbol{\sigma}_f - E_0 \boldsymbol{\varepsilon}_f) E_\varepsilon}} \dot{\boldsymbol{\sigma}}_f + E_0 \boldsymbol{\varepsilon}_f + \left(\eta + \frac{\eta E_0}{2\sqrt{(\boldsymbol{\sigma}_f - E_0 \boldsymbol{\varepsilon}_f) E_\varepsilon}} + \right) \dot{\boldsymbol{\varepsilon}}_f & \text{for} \quad \boldsymbol{\varepsilon}_f > 0 \\ 0 & \text{for} \quad \boldsymbol{\varepsilon}_f \leq 0 \end{cases} \tag{S7}$$

where $\boldsymbol{\sigma}_f$ and $\boldsymbol{\varepsilon}_f$ are the fibril stress and strain, and $\dot{\boldsymbol{\sigma}}_f$ and $\dot{\boldsymbol{\varepsilon}}_f$ are the fibril stress and strain rates. The collagen fibril stress of meniscus was formulated as [30]:

$$\boldsymbol{\sigma}_f = \begin{cases} E_0 \boldsymbol{\varepsilon}_f + \frac{1}{2} E_\varepsilon \boldsymbol{\varepsilon}_f^2 & \text{for} \quad \boldsymbol{\varepsilon}_f > 0 \\ 0 & \text{for} \quad \boldsymbol{\varepsilon}_f \leq 0 \end{cases} \tag{S8}$$

The collagen fibrillar network consisted of primary and secondary fibrils [17]. The primary collagen fibrils start from the subchondral bone and split up to the superficial zone with the arcade structure [31], while the secondary fibrils are less organized and were considered in 13 different random orientations [17]. Consequently, defining C as the amount of the primary fibrils with respect to the secondary fibrils, the stresses are given by [17]:

$\boldsymbol{\sigma}_{f,p} = C\boldsymbol{\sigma}_f$ (S9.a)

$\boldsymbol{\sigma}_{f,s} = \boldsymbol{\sigma}_f$ (S9.b)

Table S2. Material parameters for the knee joint cartilages and menisci [16–19]

Material parameter	Description	Femoral cartilage	Tibial cartilage	Patellar cartilage	Menisci
E_m (MPa)	Young modulus of the non-fibrillar matrix	0.215	0.106	0.505	0.5
v_m	Poisson's ratio of the non-fibrillar matrix	0.15	0.15	0.15	0.36
k_0 ($\times 10^{15}\ m^4/N.s$)	Initial permeability	6	18	1.9	1.25
M	Exponential term of strain-dependent permeability	5.09	15.64	15.93	5.09
η (MPa. s)	Viscosity coefficient of fibrils	1062	1062	1062	-
E_f^0 (MPa)	Initial fibril network modulus	0.92	0.18	1.88	28
E_f^ε (MPa)	Strain-dependent network modulus	150	23.6	597	-
C	The ratio of primary collagen fibers to secondary collagen fibers	12.16	12.16	12.16	12.16
$n_{f,eq}$	Fluid fraction as a function of d_n (normalized cartilage depth from the surface)	0.85-0.15d_n	0.85-0.15d_n	0.85-0.15d_n	0.72

2.2.2. Material properties the knee ligaments

Wan et al. [32] studied the effect of variation in the anterior cruciate ligament (ACL) constitutive model on the knee joint kinematics and the ACL biomechanics. They evaluated three different material models for ACL; isotropic hyperelastic, transversely isotropic hyperelastic with neo-Hookean ground matrix, and transversely isotropic hyperelastic with a nonlinear ground matrix. The material properties of the three ACL models were extracted from the same experimental data. Results of that study [32] showed that variation in the ACL constitutive model influences the knee kinematics; however, different ACL material models did not affect the estimated forces in the ACL. They concluded that the transversely isotropic hyperelastic model with nonlinear ground matrix produces the best match with experiments and that the ligament constitutive model should be chosen carefully even if the material properties of different constitutive models are obtained by fitting the models to the same experimental data [32]. Nonetheless, Wan et al. [32–34] did not evaluate how variation in the ACL constitutive model might affect e.g. biomechanics of knee cartilages. Furthermore, they did not compare their results against a knee model with ACL modeled by nonlinear springs. In fact,

the spring model we used is also nonlinear, includes compression-tension nonlinearity and has different properties in horizontal (along the length) and vertical (perpendicular to the fibrils/springs) directions.

Naghibi Beidokhti et al. [35] compared experimental data with two knee joint models, one with a nonlinear spring model and the other with a transversely isotropic continuum (3D) model of knee ligaments. They concluded that the knee model with ligaments as springs could estimate knee joint kinematics acceptably [35]. In addition, they presented comparable contact pressures on the tibial cartilage between the experiments and the estimated results of the knee model with adjusted nonlinear spring ligaments. However, they did not evaluate how variation in the parameters of the ligament constitutive models affects mechanical responses of cartilages such as fluid pressure, fibril strain, and internal stress.

In another study conducted by Orozco et al. [36], the knee model with nonlinear spring ligaments was compared against those with elastic, hyperelastic, porohyperelastic, and fibril-reinforced porohyperelastic material models of ligaments. In addition, they adjusted the material parameters of the spring model to evaluate if the knee model with the adjusted properties can replicate the response of the knee model with fibril-reinforced porohyperelastic ligaments. The joint contact force, contact pressure, and fibril strain within the tibial cartilage during the stance phase of the gait were compared between different ligament models. They concluded that the knee model including adjusted spring ligaments can reproduce similar (and even partly identical) results with the knee model utilizing fibril-reinforced porohyperelastic ligaments [36]. Yet, the segmentation, model creation, and running the 3D continuum models takes considerably more time and effort than the nonlinear spring model [36].

According to these findings and considering the main aim of the study being to develop a novel muscle force driven FE model, we selected the nonlinear spring ligament model for our study to have sufficient accuracy in the estimated parameters while keeping the computational demand reasonable.

2.3. Loading and boundary conditions

Loading inputs to the FE model, obtained from the MS model, consisted of the knee flexion angle, the muscle force vectors for each considered muscle, the residual force passing through the knee joint, and the knee abduction-adduction and internal-external moments (Fig. 2). The JCF estimated by the MS model is a combination of the 1) muscle forces, 2) inertial forces due

to the accelerations, and 3) the internal forces (excluding muscle forces) generated due to the external forces (which here is the ground reaction force). Since we applied muscle forces directly to the model, the muscle forces were subtracted from the total JCF. Thus, the inertial forces and the internal forces (which we have named as residual force passing through the joint), were imported additionally to the FE model (Fig. 2d). Finally, the residual force vector, external joint moments, and the knee flexion angle were applied into the reference point of the femur. This reference point was defined as the middle of the lateral and medial femoral condyles, and all the femoral nodes on the cartilage-bone interface were coupled to this reference point [37].

To keep the MS and FE models identical, muscle insertion points as well as muscle moment arms were imported from the MS model to the FE model. One end of each muscle was coupled to the reference point of the femur and the other end was free in space to apply the muscle force vector including both the muscle force and its direction (Fig. 2a). Consequently, each muscle generates moments on the knee joint. The external flexion-extension moment would be counterbalanced by the moment generated by muscles. In contrary, the external abduction-adduction and internal-external moment on the knee joint would not be counterbalanced by the moment generated by muscles, due to the 1 DOF knee joint in the MS model. Thus, the residual moment on the knee joint, which is the vector sum of the generated moment by muscles and the external moments applied on the knee joint, were counterbalanced by ligament. Consequently, each ligament applies a moment equal to the cross product of its moment arm and its passive force. As a result, the femur undergoes mediolateral and anteroposterior translations as well as abduction-adduction and internal-external rotations to provide the required strain in ligaments (ε in the equation 1) which generates the residual JCF forces and moments. Indeed, these residuals led to the secondary kinematics estimation by the FE model. Following, more details are presented.

The tibiofemoral joint has 1 degree of freedom (DOF) in the MS model (no ligaments are included). The inverse dynamics is performed in OpenSim to estimate the net moment at each joint. For the rotational DOFs of the model, we can write:

$$M\ddot{q} + C(q,\dot{q}) + G(q) + \tau_{ext} = \tau \,, \qquad \text{(S10)}$$

where M is the mass matrix of the body segments, q is the vector of the generalized coordinates (e.g. knee flexion angle), C is the Coriolis and centrifugal effects, G is the vector of the moment generated by the gravitational forces, τ_{ext} is the external moments applied to the body (i.e. the moment applied on the foot by the ground reaction force), and τ is the

required moment at each joint to produce the measured kinematics of the body (e.g. knee flexion-extension moment). Internal forces (R_{fs}, R_{ts}, etc.) have been assumed to pass through the joint centers. Thus, they do not generate moments around the center of the joints.

The inverse dynamics (equation S10) is solved to calculate the joint moments. Following the inverse dynamics, muscle forces are estimated to provide the required moments at each joint of the model, τ in equation S10, as:

$$\tau_j = \sum r_i^m \times F_i^m, \qquad \text{(S11)}$$

where r_i^m is the moment arm of the ith muscle around the jth DOF of the model, and F_i^m is the force generated by the ith muscle acting on the jth DOF of the MS model.

Considering Fig. S1 (the free-body diagram of the foot and shank), Newton's second law at the knee joint could be written as:

$$JCF_{knee,tot} = R_{ts} = Ma + R_{fs} + \sum F_i^{m,knee} - \sum F_k^{m,ankle} - W, \qquad \text{(S12)}$$

where $JCF_{knee,tot}$ is the total joint contact force (JCF) estimated by the MS model (equal to the internal forces between the shank and the thigh), Ma represents the inertial force of the shank due to linear acceleration, R_{fs} is the force applied from the foot to the shank, $\sum F_i^{m,knee}$ is the sum of muscle forces passing through the knee joint, $\sum F_k^{m,ankle}$ is the sum of muscle forces passing through the ankle joint, and W is the weight of the shank. Note that the effect of the GRF is implicitly included in equation S12 via R_{fs}. We should mention that all the equations S10 to S12 are solved in the OpenSim software [38].

The forces of the muscles acting on the knee joint ($\sum F_i^{m,knee}$, equation S12) were imported into the FE model of the study for each muscle, separately. Thus, the remaining terms of the equation S.12 should be imported into the FE model. We named these remaining terms a residual force passing through the knee joint (Fig. 2d) and it was calculated as:

$$F_{residual,knee} = JCF_{knee,tot} - \sum F_i^{m,knee} = Ma + R_{fs} - \sum F_k^{m,ankle} - W, \qquad \text{(S13)}$$

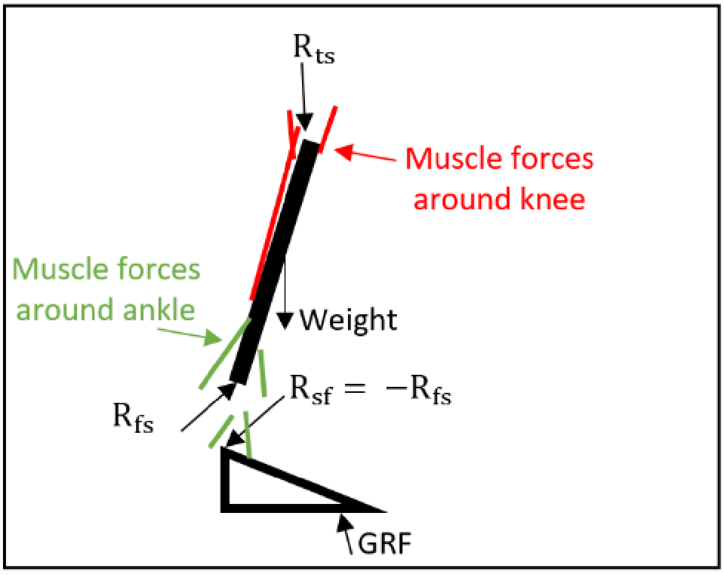

Figure S1. The free-body diagram of the foot and shank. R_{fs} is the internal force applied from the foot to the shank, R_{sf} is the internal force applied from the shank to the foot (which is equal and opposite to the R_{fs}), and R_{ts} is the internal force applied from the thigh to the shank.

Nonetheless, the joint has 6 DOFs in the finite element model (indeed 5 unconstrained DOFs, since the flexion angle is used to drive the model). Increasing the DOFs in the FE model brings the secondary kinematics to the knee joint and ligaments, as passive elements, stabilize the joint in those directions. Thus, in the FE model, the ligament forces will be added to the equation S12 and the total JCF would consist of:

$$JCF_{FE\ model,tot} = Ma + R_{fs} + \sum F_i^{m,knee} - \sum F_k^{m,ankle} - W + \sum F_j^{l}, \qquad (S14)$$

where F_j^l is the passive force in each ligament.

The flexion-extension external moment in the MS model is counterbalanced by the muscles. Thus, we can re-write equation S11 for the knee joint as (only for flexion-extension, since the joint has 1 DOF in the MS model):

$$M_{flex/ext} = \sum r_i^{m,knee} \times F_i^{m,knee}, \qquad (S15)$$

where $r_i^{m,knee}$ is the moment arm of the ith muscle. Like the force equation (equation S14), for the 6 DOFs tibiofemoral joint in the FE model, we can write:

$$M_k = \sum r_i^m \times F_i^m + \sum r_j^l \times F_j^l, \qquad (S16)$$

with M_k being the external moment on the knee joint (adduction/abduction, and internal/external) and r_j^l being the moment arm of the jth ligament (including ACL, PCL, LCL, and MCL bundles). The term "$\sum r_j^l \times F_j^l$" is called "residual moment in the knee joint".

Thus, the femur undergoes mediolateral and anteroposterior translations as well as adduction-abduction and internal-external rotations to provide the required strain in ligaments (ε in the equation 1 of the manuscript) which counterbalance external forces and moments. The word "residual" is used since those forces and moments are counterbalanced by the passive forces of ligaments.

Both the femur and the patella had six DOFs (3 translations and 3 rotations), while the bottom of the tibial cartilage was fixed in all directions. The initial condition of the FE model was set to heel strike and the complete stance phase of gait was simulated using (quasi-static) soils consolidation analysis of the Abaqus software.

2.4. Analysis

Knee joint secondary kinematics, JCF, contact area between tibial and femoral cartilages as well as tibial cartilage responses consisting of maximum principal stress, fluid pressure, and fibril strain were calculated by the FE model. Cartilage responses were reported as averaged values over the cartilage-to-cartilage contact area. To calculate the average of each parameter, first all the nodes of tibial cartilage in contact with femoral cartilage were selected in each time point of loading. Then the sum of nodal values of the parameter of interest was calculated and divided by the number of nodes in the contact area for that time increment.

2.5. Ligament sensitivity analysis

Since the magnitude of ligament pre-strain, in addition to muscle contributions, is one of the major uncertainties in the knee modeling and may significantly affect kinematics and the JCF on the joint surfaces [35,39,40], we performed a sensitivity study of the effect of pre-strain in ACL, PCL, LCL, and MCL on kinematics, kinetics and tissue responses. Table 1 (see the manuscript) shows the range of the used pre-strain values. The reference pre-strain values were obtained from the literature [41]. We generated 5 different models for each ligament (20 models in total) with -10 % to +10 % change from the reference pre-strain values. The pre-strain values of the other three ligaments were set to the reference values, while only the pre-strain of the ligament of interest was changed in each simulation.

Results

More results of ligament pre-strains sensitivity analysis including knee joint mediolateral translation (Fig. S2), and fluid pressure within the tibial cartilage (Fig. S3) have been presented here.

Discussion

The second aim of the study was to evaluate the role of each ligament pre-strain on the knee joint mechanics as one of the important uncertainties in modeling. Here, we have provided more explanations on the consistency of the current study with previous studies. Our results showed that ligament pre-strains altered both kinematics and contact parameters of the joint. Decreasing the pre-strain in the ACL and PCL restrain the anteroposterior (Fig. 7) and mediolateral (Fig. S.2) translations and internal/external rotations (Fig. 8) of the femur in relation to the tibia, which has been addressed in cadaveric studies as well [42,43]. The results showed that more pre-strained ACL reduces femur rotations and translations at heel strike and toe-off while PCL mostly decreases the range of motions and rotations of the femur during the stance phase, especially at midstance (Figs. 7 and 8).

Previous studies [44,45] reported that changes in the ACL pre-strain do not influence rotations of the femur in relation to the tibia as the knee is flexed in midstance, which is consistent with our results (Figs. 7 and 8). Our ligament sensitivity study showed that MCL is the only ligament which moves the JCF to the medial side by tightening the ligament bundle (Fig. S2), which agrees with previous studies [46,47].

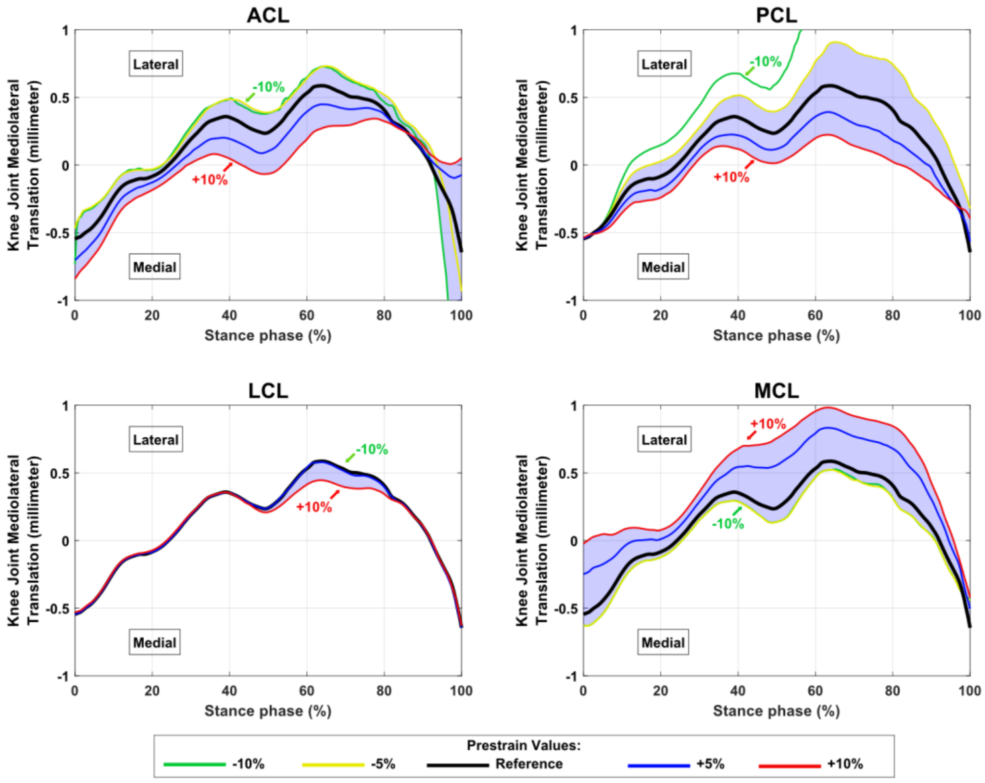

Figure S2. Knee joint mediolateral translation calculated by the FE model with different pre-strains of ACL, PCL, LCL, and MCL bundles.

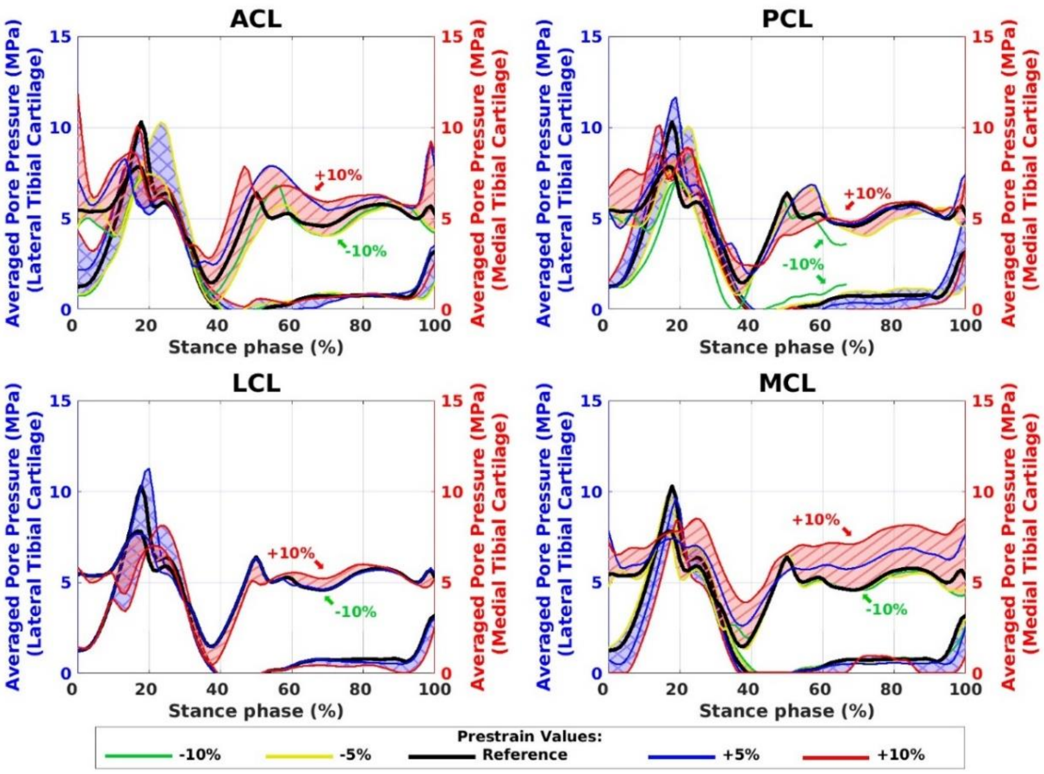

Figure S3. Average pore pressure in tibial cartilage inside the contact area with different pre-strains of ACL, PCL, LCL, and MCL bundles. The blue shaded areas (with crosslines) show the values on the lateral tibial cartilage (left axis) and the red shaded areas (diagonal lines) show the values on the medial tibial cartilage (right axes).

References

1. Hermens, H. J. *et al.* European Recommendations for Surface ElectroMyoGraphy. *Roessingh Res. Dev.* 8–11 (1999). doi:10.1016/S1050-6411(00)00027-4

2. Halonen, K. S. *et al.* Workflow assessing the effect of gait alterations on stresses in the medial tibial cartilage - Combined musculoskeletal modelling and finite element analysis. *Sci. Rep.* **7**, 1–14 (2017).

3. Liukkonen, M. K. *et al.* Evaluation of the Effect of Bariatric Surgery-Induced Weight Loss on Knee Gait and Cartilage Degeneration. *J. Biomech. Eng.* **140**, (2018).

4. Kłodowski, A. *et al.* Merge of motion analysis, multibody dynamics and finite element method for the subject-specific analysis of cartilage loading patterns during gait: differences between rotation and moment-driven models of human knee joint. *Multibody Syst. Dyn.* **37**, 271–290 (2016).

5. Marouane, H., Shirazi-Adl, A. & Adouni, M. 3D active-passive response of human

knee joint in gait is markedly altered when simulated as a planar 2D joint. *Biomech. Model. Mechanobiol.* **16**, 693–703 (2017).

6. Adouni, M. & Shirazi-Adl, A. Partitioning of knee joint internal forces in gait is dictated by the knee adduction angle and not by the knee adduction moment. *J. Biomech.* **47**, 1696–1703 (2014).

7. Adouni, M., Shirazi-Adl, A. & Shirazi, R. Computational biodynamics of human knee joint in gait: From muscle forces to cartilage stresses. *J. Biomech.* **45**, 2149–2156 (2012).

8. Lenhart, R. L., Kaiser, J., Smith, C. R. & Thelen, D. G. Prediction and validation of load-dependent behavior of the tibiofemoral and patellofemoral joints during movement. *Ann. Biomed. Eng.* **43**, 2675–2685 (2015).

9. Navacchia, A., Hume, D. R., Rullkoetter, P. J. & Shelburne, K. B. A computationally efficient strategy to estimate muscle forces in a finite element musculoskeletal model of the lower limb. *J. Biomech.* **84**, 94–102 (2019).

10. Mantoan, A. *et al.* MOtoNMS: A MATLAB toolbox to process motion data for neuromusculoskeletal modeling and simulation. *Source Code Biol. Med.* **10**, 12 (2015).

11. Marra, M. A. *et al.* A subject-specific musculoskeletal modeling framework to predict in vivo mechanics of total knee arthroplasty. *J. Biomech. Eng.* **137**, 020904 (2015).

12. Delp, S. L. *et al. OpenSim: Open-Source Software to Create and Analyze Dynamic Simulations of Movement*. *Biomedical Engineering, IEEE Transactions on* **54**, (2007).

13. Thelen, D. G., Anderson, F. C. & Delp, S. L. Generating dynamic simulations of movement using computed muscle control. *J. Biomech.* **36**, 321–328 (2003).

14. van Arkel, R. J., Modenese, L., Phillips, A. T. M. & Jeffers, J. R. T. Hip abduction can prevent posterior edge loading of hip replacements. *J. Orthop. Res.* **31**, 1172–1179 (2013).

15. Halonen, K. S. *et al.* Importance of Patella, Quadriceps Forces, and Depthwise Cartilage Structure on Knee Joint Motion and Cartilage Response During Gait. *J. Biomech. Eng.* **138**, 71002–71011 (2016).

16. Julkunen, P., Kiviranta, P., Wilson, W., Jurvelin, J. S. & Korhonen, R. K.

Characterization of articular cartilage by combining microscopic analysis with a fibril-reinforced finite-element model. *J. Biomech.* **40**, 1862–70 (2007).

17. Wilson, W., van Donkelaar, C. C., van Rietbergen, B., Ito, K. & Huiskes, R. Stresses in the local collagen network of articular cartilage: a poroviscoelastic fibril-reinforced finite element study. *J. Biomech.* **37**, 357–366 (2004).

18. Dabiri, Y. & Li, L. P. Influences of the depth-dependent material inhomogeneity of articular cartilage on the fluid pressurization in the human knee. *Med. Eng. Phys.* **35**, 1591–1598 (2013).

19. Makris, E. A., Hadidi, P. & Athanasiou, K. A. The knee meniscus: Structure–function, pathophysiology, current repair techniques, and prospects for regeneration. *Biomaterials* **32**, 7411–7431 (2011).

20. Böttcher, P., ZEISSLER, M., MAIERL, J., GREVEL, V. & OECHTERING, G. Mapping of split-line pattern and cartilage thickness of selected donor and recipient sites for autologous osteochondral transplantation in the canine stifle joint. *Vet. Surg.* **38**, 696–704 (2009).

21. Leo, B. M., Turner, M. A. & Diduch, D. R. Split-line pattern and histologic analysis of a human osteochondral plug graft. *Arthrosc. J. Arthrosc. Relat. Surg.* **20**, 39–45 (2004).

22. Goodwin, D. W. *et al.* Macroscopic Structure of Articular Cartilage of the Tibial Plateau: Influence of a Characteristic Matrix Architecture on MRI Appearance. *Am. J. Roentgenol.* **182**, 311–318 (2004).

23. Below, S., Arnoczky, S. P., Dodds, J., Kooima, C. & Walter, N. The split-line pattern of the distal femur: A consideration in the orientation of autologous cartilage grafts. *Arthrosc. J. Arthrosc. Relat. Surg.* **18**, 613–617 (2002).

24. Mononen, M. E. *et al.* Effect of superficial collagen patterns and fibrillation of femoral articular cartilage on knee joint mechanics—A 3D finite element analysis. *J. Biomech.* **45**, 579–587 (2012).

25. Wilson, W. A Comparison Between Mechano-Electrochemical and Biphasic Swelling Theories for Soft Hydrated Tissues. *J. Biomech. Eng.* **127**, 158 (2005).

26. Lai, W. M., Mow, V. C. & Roth, V. Effects of Nonlinear Strain-Dependent

Permeability and Rate of Compression on the Stress Behavior of Articular Cartilage. *J. Biomech. Eng.* **103**, 61 (1981).

27. van der Voet, A. A comparison of finite element codes for the solution of biphasic poroelastic problems. *Proc. Inst. Mech. Eng. H.* **211**, 209–11 (1997).

28. Lipshitz, H., Etheredge, R. 3rd & Glimcher, M. J. In vitro wear of articular cartilage. *JBJS* **57**, (1975).

29. Wilson, W., van Donkelaar, C. C., van Rietbergen, B., Ito, K. & Huiskes, R. Erratum to "Stresses in the local collagen network of articular cartilage: a poroviscoelastic fibril-reinforced finite element study" [Journal of Biomechanics 37 (2004) 357–366] and "A fibril-reinforced poroviscoelastic swelling model for articular cartil. *J. Biomech.* **38**, 2138–2140 (2005).

30. Danso, E. K., Honkanen, J. T. J., Saarakkala, S. & Korhonen, R. K. Comparison of nonlinear mechanical properties of bovine articular cartilage and meniscus. *J. Biomech.* **47**, 200–206 (2014).

31. Benninghoff, A. Form und Bau der Gelenkknorpel in ihren Beziehungen zur Funktion. *Zeitschrift für Zellforsch.* **2**, 783–862 (1925).

32. Wan, C., Hao, Z. & Wen, S. The effect of the variation in ACL constitutive model on joint kinematics and biomechanics under different loads: A finite element study. *J. Biomech. Eng.* **135**, (2013).

33. Wan, C., Hao, Z. & Wen, S. A comparison of material characterizations in frequently used constitutive models of ligaments. *Int. j. numer. method. biomed. eng.* **30**, 605–615 (2014).

34. Wan, C., Hao, Z. & Wen, S. A review on research on development of ligament constitutive relations on macro, meso, and micro levels. *Acta Mech. Solida Sin.* **26**, 331–343 (2013).

35. Naghibi Beidokhti, H. *et al.* The influence of ligament modelling strategies on the predictive capability of finite element models of the human knee joint. *J. Biomech.* **65**, 1–11 (2017).

36. Orozco, G. A., Tanska, P., Mononen, M. E., Halonen, K. S. & Korhonen, R. K. The effect of constitutive representations and structural constituents of ligaments on knee

joint mechanics. *Sci. Rep.* **8**, 2323 (2018).

37. Mononen, M. E., Jurvelin, J. S. & Korhonen, R. K. Implementation of a gait cycle loading into healthy and meniscectomised knee joint models with fibril-reinforced articular cartilage. *Comput. Methods Biomech. Biomed. Engin.* **18**, 141–152 (2015).

38. Steele, K. M., DeMers, M. S., Schwartz, M. H. & Delp, S. L. Compressive tibiofemoral force during crouch gait. *Gait Posture* **35**, 556–560 (2012).

39. Mesfar, W. & Shirazi-Adl, A. Biomechanics of changes in ACL and PCL material properties or prestrains in flexion under muscle force-implications in ligament reconstruction. *Comput. Methods Biomech. Biomed. Engin.* **9**, 201–209 (2006).

40. Halonen, K. S. *et al.* Optimal graft stiffness and pre-strain restore normal joint motion and cartilage responses in ACL reconstructed knee. *J. Biomech.* **49**, 2566–2576 (2016).

41. Blankevoort, L. & Huiskes, R. Ligament-bone interaction in a three-dimensional model of the knee. *J. Biomech. Eng.* **113**, 263–9 (1991).

42. Fukubayashi, T., Torzilli, P. A., Sherman, M. F. & Warren, R. *An in vitro biomechanical evaluation of anterior-posterior motion of the knee. Tibial displacement, rotation, and torque. The Journal of bone and joint surgery. American volume* **64**, (1982).

43. Melby, A., Noble, J. S., Askew, M. J., Boom, A. A. & Hurst, F. W. The effects of graft tensioning on the laxity and kinematics of the anterior cruciate ligament reconstructed knee. *Arthrosc. J. Arthrosc. Relat. Surg.* **7**, 257–266 (1991).

44. Markolf, K. L., Mensch, J. S. & Amstutz, H. C. Stiffness and laxity of the knee--the contributions of the supporting structures. A quantitative in vitro study. *J. Bone Joint Surg. Am.* **58**, 583–94 (1976).

45. Smith, C. R., Lenhart, R. L., Kaiser, J., Vignos, M. F. & Thelen, D. G. Influence of Ligament Properties on Tibiofemoral Mechanics in Walking. *J. Knee Surg.* **29**, 99–106 (2016).

46. Crottet, D. *et al.* Ligament balancing in TKA: Evaluation of a force-sensing device and the influence of patellar eversion and ligament release. *J. Biomech.* **40**, 1709–1715 (2007).

47. Smith, C. R., Vignos, M. F., Lenhart, R. L., Kaiser, J. & Thelen, D. G. The Influence of Component Alignment and Ligament Properties on Tibiofemoral Contact Forces in Total Knee Replacement. *J. Biomech. Eng.* **138**, 21010–21017 (2016).
